# Interplay of Trypanosome Lytic Factor and innate immune cells in the resolution of cutaneous *Leishmania* infection

**DOI:** 10.1371/journal.ppat.1008768

**Published:** 2021-09-24

**Authors:** Jyoti Pant, Marie Samanovic, Maria T. Nelson, Mert K. Keceli, Joseph Verdi, Stephen M. Beverley, Jayne Raper

**Affiliations:** 1 Department of Biology, Hunter College, City University of New York, New York, New York, United States of America; 2 Molecular, Cellular and Developmental biology, The Graduate Center, City University of New York, New York, New York, United States of America; 3 Medical Parasitology, New York University Langone Medical Center, New York, New York, United States of America; 4 Department of Molecular Microbiology, Washington University School of Medicine, St. Louis, Missouri, United States of America; University of Manitoba, CANADA

## Abstract

Trypanosome Lytic Factor (TLF) is a primate-specific high-density lipoprotein (HDL) complex that, through the cation channel-forming protein apolipoprotein L-1 (APOL1), provides innate immunity to select kinetoplastid parasites. The immunoprotective effects of TLF have been extensively investigated in the context of its interaction with the extracellular protozoan *Trypanosoma brucei brucei*, to which it confers sterile immunity. We previously showed that TLF could act against an intracellular pathogen *Leishmania*, and here we dissected the role of TLF and its synergy with host-immune cells. *Leishmania major* is transmitted by Phlebotomine sand flies, which deposit the parasite intradermally into mammalian hosts, where neutrophils are the predominant phagocytes recruited to the site of infection. Once in the host, the parasites are phagocytosed and shed their surface glycoconjugates during differentiation to the mammalian-resident amastigote stage. Our data show that mice producing TLF have reduced parasite burdens when infected intradermally with metacyclic promastigotes of *L*. *major*, the infective, fly-transmitted stage. This TLF-mediated reduction in parasite burden was lost in neutrophil-depleted mice, suggesting that early recruitment of neutrophils is required for TLF-mediated killing of *L*. *major*. *In vitro* we find that only metacyclic promastigotes co-incubated with TLF in an acidic milieu were lysed. However, amastigotes were not killed by TLF at any pH. These findings correlated with binding experiments, revealing that labeled TLF binds specifically to the surface of metacyclic promastigotes, but not to amastigotes. Metacyclic promastigotes of *L*. *major* deficient in the synthesis of surface glycoconjugates LPG and/or PPG (*lpg1*^*-*^ and *lpg5A*^*-*^*/lpg5B*^*-*^ respectively) whose absence mimics the amastigote surface, were resistant to TLF-mediated lysis. We propose that TLF binds to the outer surface glycoconjugates of metacyclic promastigotes, whereupon it kills the parasite in the acidic phagosome of phagocytes. We hypothesize that resistance to TLF requires shedding of the surface glycoconjugates, which occurs several hours after phagocytosis by immune cells, creating a relatively short-lived but effective window for TLF to act against *Leishmania*.

## Author summary

Leishmaniasis, the disease caused by parasites of the genus *Leishmania*, can be divided into cutaneous, muco-cutaneous and visceral leishmaniasis depending on the parasite species and the clinical outcome of the disease. Of the three, cutaneous leishmaniasis is the most common form, which is usually characterized by a localized lesion due to the infection of immune cells, primarily dermal and lymph node-resident macrophages. The time between infection and lesion appearance ranges from weeks to years, while some individuals never develop lesions. The length of this subclinical stage of leishmaniasis depends on a variety of factors: parasite virulence, infectious dose, and the host immune response. Therefore, it remains crucial to develop our understanding of each component of the host-parasite interface and assess the role that each component plays in the clinical outcome. Here, we analyze the interaction between *L*. *major*, a cutaneous strain, and the host innate immune factor Trypanosome Lytic Factor (TLF), a sub-class of circulating High-Density Lipoprotein (HDL). TLF provides sterile immunity to most extracellular African Trypanosomes by osmotically lysing the parasites. Lysis is driven by the primate specific protein apolipoprotein L-1 (APOL1), a cation channel-forming protein that is activated by a series of pH-dependent conformational changes. APOL1 inserts into cellular membranes at acidic pH and forms a closed ion channel that subsequently opens when re-exposed to neutral pH, resulting in ion flux.

Using transgenic mice producing primate TLF, we show that both human and baboon TLFs ameliorate cutaneous *Leishmania major* infection and that this reduction in parasite burden correlates with: 1. infectious dose of metacyclic promastigotes 2. the concentration of circulating TLF in plasma and 3. early recruitment of neutrophils at the site of infection. Our results show that the acidification step is essential for TLF-mediated lysis of axenic metacyclic promastigotes of *Leishmania in vitro*. The susceptibility of metacyclic promastigotes to TLF-mediated lysis is governed by the surface glycoconjugates of *Leishmania*. We find that surface glycoconjugate-deficient *Leishmania* are resistant to TLF-mediated killing. Based on these data, we conclude that the shedding of surface glycoconjugates while transitioning from metacyclic promastigotes to amastigotes, results in parasite resistance to TLF-mediated lysis. Whether TLF is effective at killing metacyclic promastigotes of other experimentally tractable *Leishmania* sp., such as *L*. *infantum* and *L*. *donovani*, which have different surface glycoconjugate structures is yet to be tested. Our data raise the possibility that TLF may have lytic activity against a broader range of pathogens such as bacteria, viruses, fungi and parasites with surface glycoconjugates that transit through intracellular acidic compartments.

## Introduction

*Leishmania* sp. are intracellular eukaryotic parasites responsible for a spectrum of diseases ranging from mild cutaneous lesions to fatal visceral infections. The parasites are transmitted to humans by the bite of a sand fly vector, wherein the infective, motile, flagellated metacyclic promastigote form is deposited intradermally into the host. Therein *Leishmania* are taken up by phagocytes and transform into the intracellular, aflagellate amastigote form, which go on to replicate within an acidic parasitophorous vacuole [1]. Although macrophages are the primary host cells for *Leishmania*, parasites can be phagocytosed by other immune cell types including neutrophils and dendritic cells [2–4]. The importance of neutrophils early in infection has received increasing attention as a key factor in disease progression, infiltrating the site of the bite as early as 1-hour post-infection and potentially contributing to resolution of the infection [2,3,5].

In addition to defenses associated with host cell uptake, *Leishmania* must survive against a variety of extracellular host defenses such as complement [6,7]. In 2009, our laboratory showed that trypanosome lytic factor (TLF), high-density lipoprotein (HDL) complex originally characterized for its ability to kill extracellular African trypanosomes, could also ameliorate infection by cutaneous *Leishmania* sp. [8]. TLF is produced only in humans and some higher primates and contains the primate-specific proteins haptoglobin-related protein (HPR), and apolipoprotein L-1 (APOL1), the lytic factor, a cation channel forming protein [9–14]. TLF is endocytosed by blood-circulating Trypanosomes, whereupon APOL1 encounters the acidified environment required for insertion of a closed APOL1 ion channel into the parasite endocytic membrane [13,15,16,17]. The ion channel opens upon neutralization, which occurs at the plasma membrane after membrane recycling, leading to ion flux [17], consequent ion imbalance, and ultimately colloid-osmotic lysis. Although APOL1 alone is necessary and sufficient for the trypanolytic activity of TLF [14,16,18], the presence of HPR significantly enhances trypanosome lysis by increasing the uptake of TLF by the parasites, via the haptoglobin-hemoglobin receptor (TbHpHbR) [19]. In contrast, TLF kills the intracellular metacyclic promastigotes of *L*. *major* and *L*. *amazonensis* within the phagosomes of macrophages [8]. Although APOL1 associates with and forms ion channels in cellular membranes (lipid bilayers) in a pH-dependent manner [13,17], the role of phagosomal pH changes in immune cells on the TLF-mediated killing of *Leishmania* have not been investigated. We hypothesize that acidification of phagosomes containing parasites is essential for TLF-mediated lysis of metacyclic promastigotes within these cells. Following uptake by phagocytes, however, infective *Leishmania* metacyclic promastigotes transiently delay phagosomal acidification, allowing for differentiation into the phagocyte-resident amastigote form. This mechanism is mediated primarily by the surface glycoconjugate lipophosphoglycan (LPG), which is abundant on the metacyclic promastigote cell membrane, but not that of the amastigote form [20–23]. Importantly, the amastigote forms of the parasite are resistant to TLF-mediated killing activity, the basis of which is not understood [8] but could conceivably involve these life cycle stage-associated differences in surface architecture.

In this study, we demonstrate that TLF reduces the intradermal parasite burden in transgenic mice producing human or baboon TLF and that neutrophil recruitment and acidification of infected immune cell phagosomes is essential for this TLF-mediated protection. Finally, using the glycoconjugate mutants *lpg1*^-^ and *lpg5A*^*-*^ */lpg5B*^*-*^, we show that surface glycoconjugates are required for TLF-interaction and lysis and hypothesize that shedding of the glycoconjugates not only delay acidification of the phagosomes [4,20–24], but also removes the TLF from the surface of the parasites, underlying the resistance of the amastigote form.

## Results

### Human TLF reduces the parasite burden in a natural intradermal infection by *L*. *major*

While laboratory mice are good models for *Leishmania* infection and rodents are a natural zoonotic host, they do not express TLF [8,9,16]. To circumvent this issue, we frequently use hydrodynamic gene delivery (HGD) to introduce the HPR and APOL1 genes, which are secreted and assembled on endogenous murine HDL in the blood [8,9,16]. This generates mice that transiently express human TLF. Using this robust expression system, the levels of TLF in the plasma of transfected C57/Bl6 mice after one day are similar to levels in human plasma [8]. In previous work, we then employed the classic subcutaneous footpad infection model for studying the effect of TLF on *L*. *major* [8]. Here, we instead employed the intradermal ear infection model, which more closely mimics the infection route and numbers of parasites in natural infections [2,3,25,26].

**Fig 1:**
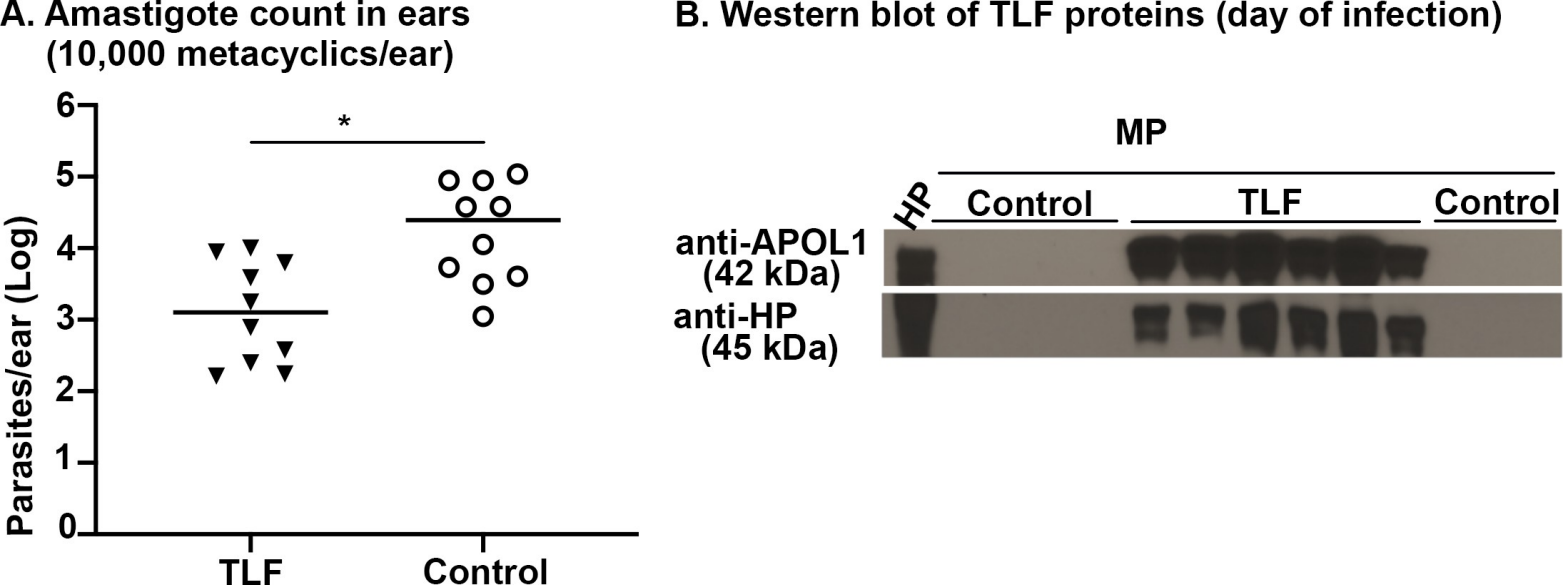
Human HDL reduces the parasite burden in a natural intradermal infection by *L*. *major*. **A.** Transiently transgenic mice were created by injecting 50μg of plasmid DNA containing human *APOL1*:*HPR* genes (TLF) in a single plasmid by hydrodynamic gene delivery (HGD) followed by infection by 10,000 metacyclic promastigotes (1 day post-HGD). Control groups were injected empty vector plasmid. Parasite burden (amastigotes) was quantified by real time PCR, 15 days post-infection. The data represent Mean ± SD of one typical experiment that has been repeated four times **p<0.001 Mann-Whitney U test, n = 5 mice (10 ears) per group, 1 mouse from each TLF and control group was euthanized before 15 days due to tail inflammation. **B.** Western Blots of plasma samples from mice on the day of infection diluted 1:40 (mouse plasma, MP) n = 6 per group were performed to assess the production of the APOL1 (42 kDa) and HPR (45 kDa) proteins. HP (human plasma) diluted 1:40 was used as positive control.

These mice produce detectable levels of the TLF proteins (APOL1 and HPR) for at least 3 days post infection (Day 2- peak time of TLF production) (S1 Fig). The mice were then injected intradermally in the ear with metacyclic promastigotes one day after HGD. Parasites were quantified in the ears 15 days post-infection by qPCR. Consistent with previous studies using the intradermal footpad model, parasites numbers were significantly reduced (*p* = 0.0153, ~12-fold reduction) relative to saline HGD controls (Fig 1A). The production of TLF proteins was confirmed in murine-plasma by western blot at the time of infection to check the success of HGD injection (Fig 1B). Thus, we observed TLF-dependent control of *L*. *major* via two infectious routes, subcutaneous [8] and intradermal herein.

### Germline targeted transgenic mice expressing baboon TLF ameliorate intradermal infection by *L*. *major*

**Fig 2:**
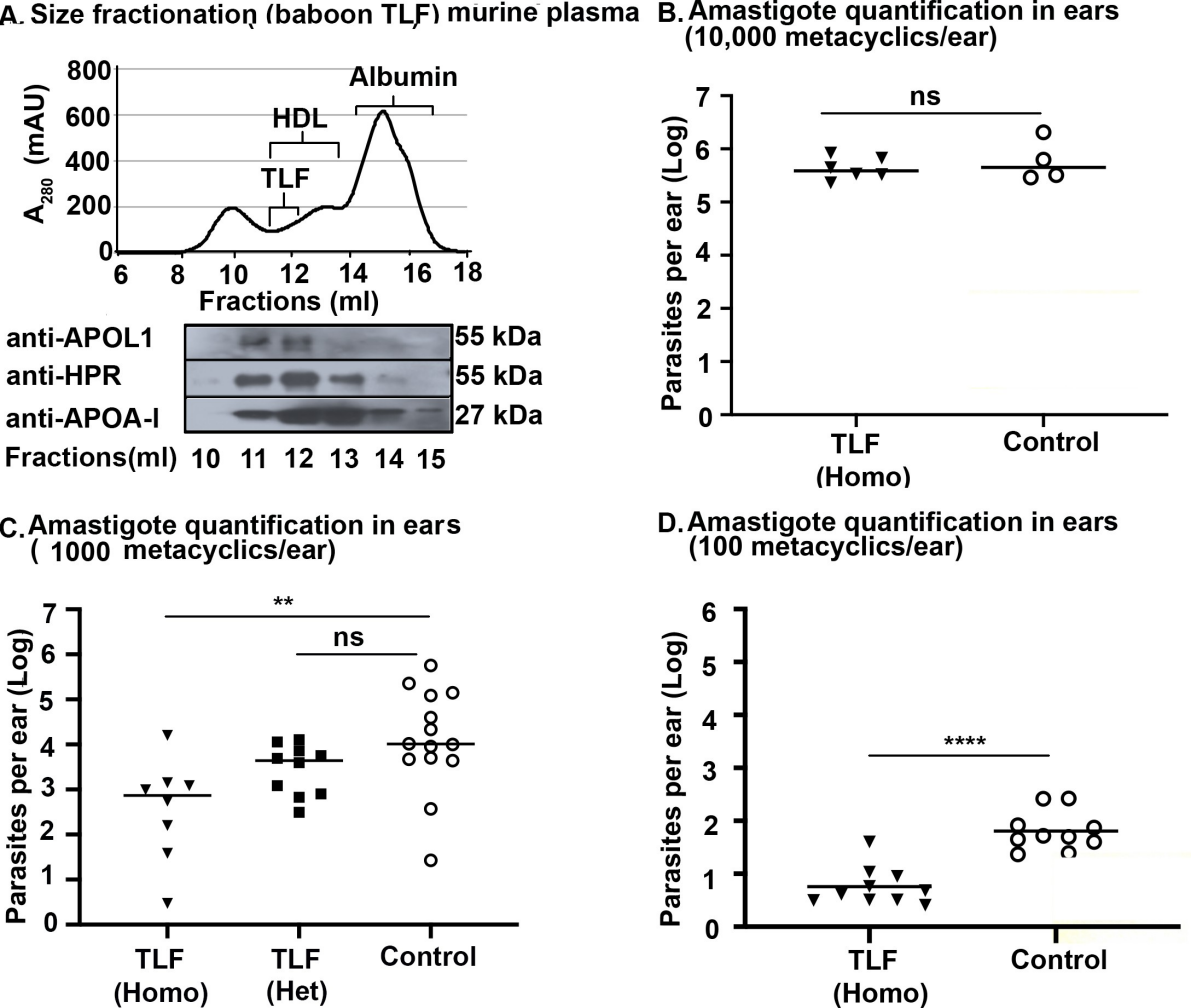
Germline targeted transgenic mice expressing baboon TLF ameliorate intradermal infection by *L*. *major*. **A.** Plasma from germline transgenic mice expressing baboon *APOL1* and *HPR* separated by size exclusion chromatography showing the protein absorbance profile (280 nm) and the western blot of the fractions (10–15) for three TLF proteins- APOL1, HPR and APOA-I. Fractions 11–14 contain HDL. **B–D.** Number of parasites in germline transgenic mice ears (Homo = homozygotes, Het = Heterozygotes) quantified by real-time PCR 15 days post-infection with: **B.** 10,000 metacyclic promastigotes n = 3 mice, 6 ears (TLF), n = 2 mice, 4 ears (control). **C.** 1000 metacyclic promastigotes, n = 4 mice, 8 ears (Homo), 5 mice, 10 ears (Het), 7 mice, 14 ears (Control). **D.** 100 metacyclic promastigotes, n = 5 mice, 10 ears per group. The data represents Mean ± SD of one typical experiment that has been repeated three times. **p<0.001, **** *p*<0.0001 and ns- non-significant, Mann-Whitney U test (Fig 2B and 2D) and ANOVA multiple comparisons with Bonferroni correction (Fig 2C) were used for statistical analysis.

While the transient HGD mouse model has many advantages, we found plasmid DNA injection by HGD was highly inflammatory and strongly increased the percentage of leukocytes (CD11b+) and neutrophils (CD11b+Ly6G+Ly6C^high^) in the blood (S1B Fig), two major cell types known to play important roles in *Leishmania* infection [2,3]. In addition, the production of the TLF-proteins in mice was transient, lasting only 3 days after hydrodynamic gene delivery (S1A Fig). To explore TLF-*Leishmania* interactions in a less inflammatory setting with sustained TLF production *in vivo*, we took advantage of a recently produced germline transgenic murine model that produces baboon TLF (we do not have germline transgenic human TLF mice). As expected, the APOL1 and HPR in these mice can be detected in lipoprotein complexes upon density gradient centrifugation and, as in primate (human and baboon) plasma, both proteins elute together with the structural protein APOA-I (present in all HDL) in ~500kDa HDL fractions after size-exclusion chromatography (fractions 11–13), indicating that a complete TLF complex was reconstituted in these mice (Fig 2A). The germline transgenic mice produce 100-fold less TLF proteins compared to HGD mice (S3A Fig). Despite reduced circulating TLF, the germline mice were resistant to the African trypanosome *T*. *b*. *brucei* (S2B Fig). Hence, we expect the TLF of these mice to restrict *Leishmania* similar to human TLF in the transiently transfected mice (Fig 1). We infected the germline transgenic mice with 10^4^ metacyclic promastigotes. However, we did not see a difference in the number of parasites (quantified by qPCR) between TLF-expressing (homozygous mice) and the control mice 15 days post-infection. In contrast, we observed a ten-fold reduction in parasite burden at an infective dose of 10^3^ metacyclic promastigotes (*p* = 0.0031) and 100 metacyclic promastigotes (*p*<0.0001) (Fig 2B–2D). Our results show that the outcome of TLF activity against *L*. *major* is governed in part by the infective dose of the parasites.

We also observed that the magnitude of any TLF-mediated protection also depends on the host’s circulating TLF/HDL concentration. Homozygous mice with two copies of *APOL1* and *HPR* produce twice as much of each of the TLF proteins (APOL1 and HPR) as heterozygous mice (S2C Fig). Accordingly, homozygous mice show a ten-fold reduction (*p* = 0.0031) in parasite burden compared to the heterozygous mice that show only a five-fold reduction (*p* = 0.2464) (Fig 2C). These results show that a threshold of circulating TLF is required for reducing the parasite burden at the site of infection. Our results suggest that both human TLF and baboon TLF can reduce the *L*. *major* burden after intradermal delivery of the parasites. We conclude that the final outcome of the disease depends on the initial dose of infecting parasites and the concentration of circulating TLF in the plasma.

### Neutrophils are required for TLF-mediated protection against *L*. *major in vivo*

**Fig 3:**
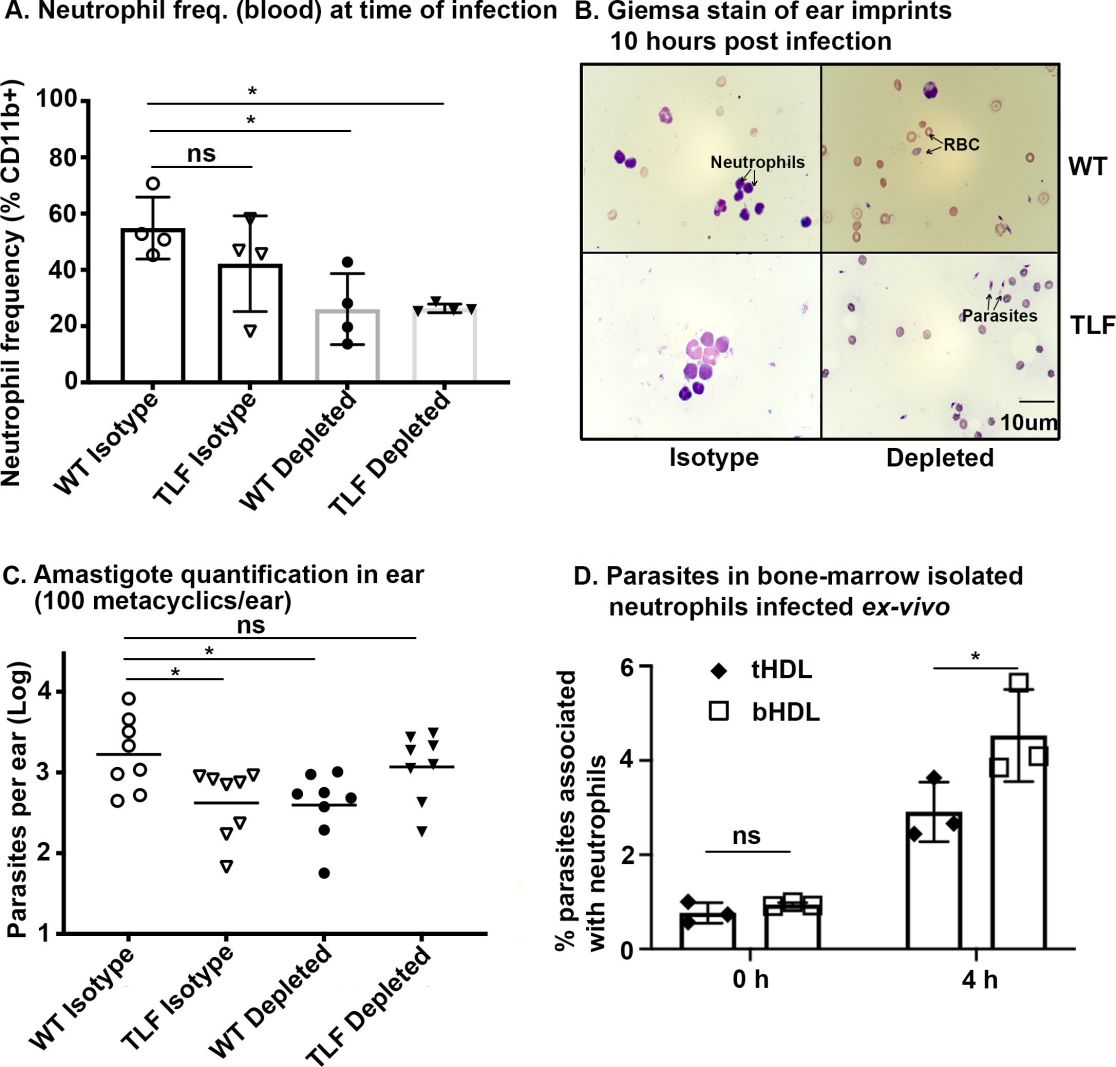
Neutrophils are required for TLF-mediated protection against *L*. *major in vivo*. Neutrophils were depleted in mice using 1 mg anti-mouse Ly6G clone 1A8 antibody (depleted) or not depleted using an isotype matched IgG2A antibody (isotype). **A.** Neutrophil frequencies were assayed 24 hours post-depletion (time of infection) by collecting blood from the tail, staining for surface markers and analyzing by flow cytometry. The bar graph depicts the neutrophil frequency (CD11b^+^Ly6G^+^Ly6C^+^). Data analyzed by Barlett test for Normality distribution and a One-way ANOVA to determine if frequency of neutrophils were different among groups (p = 0.0272). Comparison between groups was done by Tukey post hoc analysis. **B.** Giemsa stains of the ear imprints collected 10 hours post-infection from wild-type mice and TLF mice with (isotype) or without neutrophils (depleted) visualized using Nikon Eclipse 50i microscope under 100X oil immersion. The data is representative of multiple fields visualized that were repeated twice. **C**. The number of parasites quantified by real time PCR in mice ears harvested 15 days post-infection with 100 metacyclic promastigotes, n = 4 mice, 8 ears per group. The data represents Mean ± SD of one typical experiment that has been repeated twice. * *p*<0.05, variance and normality tested using Barlett test and Shapiro-Wilk normality test which were both significant. Comparison between groups was performed by non-parametric Kruskal-Wallis t-test with Bonferroni Correction. D. Bone-marrow isolated neutrophils were infected with CFSE stained metacyclic promastigotes at MOI of 3:1 and phagocytosed parasites were assayed at 2 and 4 hours post infection. The data represents Mean ± SD of one typical experiment that has been repeated four times. *p<0.01, ns- non-significant, 2-way ANOVA, multiple comparisons with Bonferroni correction.

Neutrophils are the first cells recruited and infected by metacyclic promastigotes in an intradermal infection by *L*. *major* [2,3]. To test their role in TLF-mediated protection, neutrophils were depleted from transgenic mice expressing baboon TLF (homozygotes) and control (wild-type) mice by injecting anti-mouse Ly6G 1A8 antibodies, with isotype matched IgG antibody as a control. After 24 hours, a 50% reduction of neutrophils was observed in the blood (Fig 3A). Then, mice were infected with 100 metacyclic promastigotes per ear. At 10 hours post-infection we also observed a reduction of neutrophils in the ears (site of infection) of mice injected with anti-mouse Ly6G 1A8 antibodies (Fig 3B) when observed by Giemsa stain, showing that infection did not interfere with depletion. When there was ~50% reduction in neutrophils in the blood at the time of infection (S3B Fig), there was a ~67% (p<0.0001) reduction in recruitment of neutrophils in the ear (S3B Fig).

Despite similar neutrophil numbers in wild type and TLF mice injected with isotype antibodies (p = 0.46, Fig 3A), when inoculated with 100 metacyclic promastigotes there was a ~6-fold reduction in the amastigote numbers in the ears of mice producing TLF compared to WT mice (p = 0.048, Fig 3C). Upon depletion of neutrophils with anti-mouse Ly6G 1A8 antibodies in the wild type mice (p = 0.036), we observed a ~6-fold reduction in the amastigote numbers (p = 0.041) compared to WT isotype mice (Fig 3A and 3C). In contrast, TLF mice depleted of neutrophils have no change in the amastigote numbers in the ears compared to WT isotype mice (Fig 3C).

These data suggest that while neutrophils promote infection in the absence of TLF [2,3,5], in the presence of TLF, neutrophils can act to reduce the parasite burden, suggesting a dynamic interaction between TLF, *L*. *major* metacyclic promastigotes, and neutrophils during intradermal infections. To further explore this model, we infected purified bone-marrow derived neutrophils exposed to TLF enriched human HDL (tHDL) or APOA1 enriched bovine HDL (bHDL, control), with fluorescent metacyclic promastigotes (CFSE, see Materials and Methods). We observed significantly fewer parasites (40% reduction) in the presence of TLF/tHDL compared to control bHDL at 4 hours post infection (Figs 3D and S4A). These results support the *in vivo* findings and additionally suggest there is a relatively small window for TLF to act within neutrophils further validating the dynamic interaction between TLF, metacyclic promastigotes and neutrophils.

### Acidic pH is essential for TLF-mediated lysis of *L*. *major*

**Fig 4:**
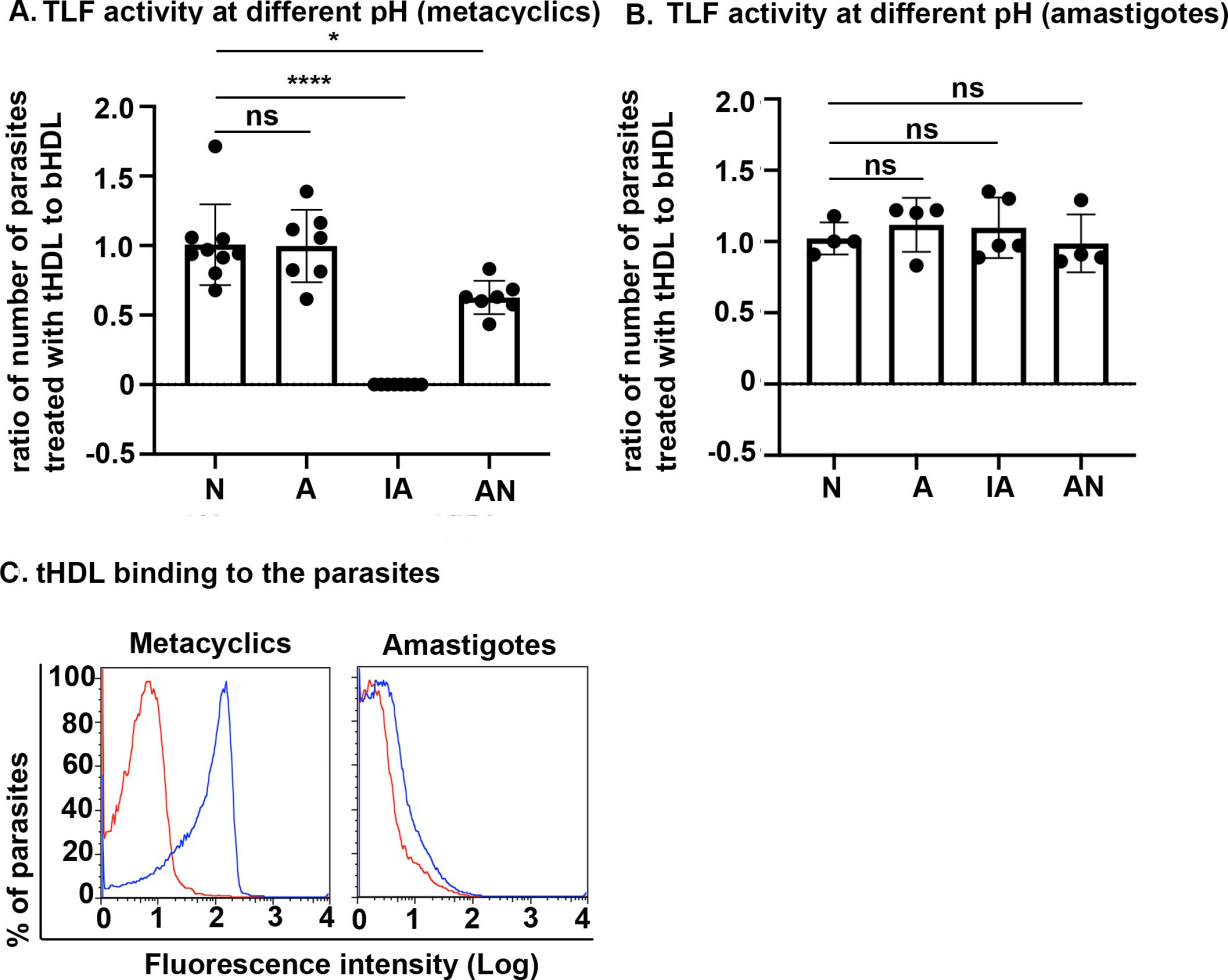
Acidic pH is essential for TLF-mediated lysis of *L*. *amazonensis*. **A**. Metacyclic promastigotes or **B.** amastigotes of *L*. *amazonensis* were treated with human HDL (1.5 mg/ml) or bovine HDL (1.5 mg/ml) and incubated in different conditions; Neutral—N pH 7.4 for 2 hours, Acidic–A pH 5.6 for 2 hours, Increased acidification- IA pH 5.6 for 1 hour followed by pH 4.5 for 1 hour, and Acid and Neutral- AN pH 5.6 for 1 hour followed by pH 7.4 for 1 hour. Parasites were microscopically counted using a hemocytometer. The data represent the means ± standard deviation of three experiments that have been combined. **** *p*<0.0001, ns- non-significant, ANOVA test, multiple comparisons with Bonferroni correction. **C.** *L*. *amazonensis* metacyclic promastigotes or axenic amastigote forms (1x10^6^/ml) were treated with 10 μg/ml of DyLight-488 labelled tHDL (TLF, blue) or not (red) for 30 min on ice. Fluorescence intensity was quantified by flow cytometry. The data represent one typical experiment repeated three times.

Based on previous results, TLF kills metacyclic promastigotes of *Leishmania* in acidic conditions in 24 hours *in vitro* and in macrophages *ex vivo* [8]. However, our neutrophil results demonstrate that the window of activity of TLF is relatively small. To further investigate the potential mechanism of lysis of the parasites within neutrophil phagosomes, we incubated metacyclic promastigotes with TLF at various pH conditions (that broadly occur in phagocytes) for two hours: Neutral, (pH 7.4 for 2 h), Acidic (pH 5.6 for 2 hours), Increased Acidification (pH 5.6 for 1 hour followed by pH 4.5 for 1 hour), Acid and Neutral (pH 5.6 for 1 hour followed by pH 7.4 for 1 hour). We used TLF resistant amastigotes [8] and TLF-lacking bovine HDL (bHDL) controls. As we cannot obtain axenic amastigotes from *L*. *major*, we used *L*. *amazonensis*, another TLF-susceptible cutaneous strain [8]. No killing of the metacyclic promastigotes was observed at any pH in the presence of bHDL control (Fig 4A). The data are expressed as a ratio of surviving parasites post human HDL (TLF)-treatment: bHDL-treatment. A ratio of one would indicate equivalent survival in both treatments, while less than one would indicate increased parasite killing by tHDL (TLF)-treatment. Metacyclic promastigotes subjected to increased acidification (Fig 4A) (IA) in the presence of tHDL were completely lysed in two hours (p<0.0001). We also observed a significant reduction (p = 0.0036) in parasite number when metacyclic promastigotes were incubated with tHDL and subjected to acidification (pH 5.6 for 1 hour) followed by neutralization (pH 7 for 1 hour) (Fig 4A) (AN). This reduction in parasite number was not observed in metacyclic promastigotes incubated with tHDL at neutral pH (N, pH 7) or acidic pH (A, pH 5.6) for 2 hours (Fig 4A). These data underline the importance of pH changes to the TLF-mediated lysis of metacyclic promastigotes within a small timeline, whereby the key acidification step likely occurs within the phagosomes of immune cells [8,20–22,24]. Consistent with our previous results, we did not observe lysis of axenic amastigotes by tHDL at any pH suggesting that axenic amastigotes are resistant to TLF activity (Fig 4B). Therefore, TLF-mediated protection is restricted to the early stage of infection when the parasite exists as a metacyclic promastigote. One notable difference between metacyclic promastigotes and amastigotes is the downregulation of many surface glycoconjugates in the amastigote form, suggesting that there could be a specific interaction between TLF and the surface of metacyclic promastigotes. Therefore, we analyzed the binding between tHDL and both metacyclic promastigotes and axenic amastigotes. We observed that both tHDL and bHDL interact with metacyclic promastigotes but not with the resistant amastigotes (Figs 4C and S5) suggesting that TLF-mediated lysis could be affected by the differences between the surface glycoconjugates of the two life-cycle stages.

### *S*urface glycoconjugate mutants of *L*. *major* are less susceptible to TLF-mediated lysis than wild-type parasites in the macrophage

**Fig 5:**
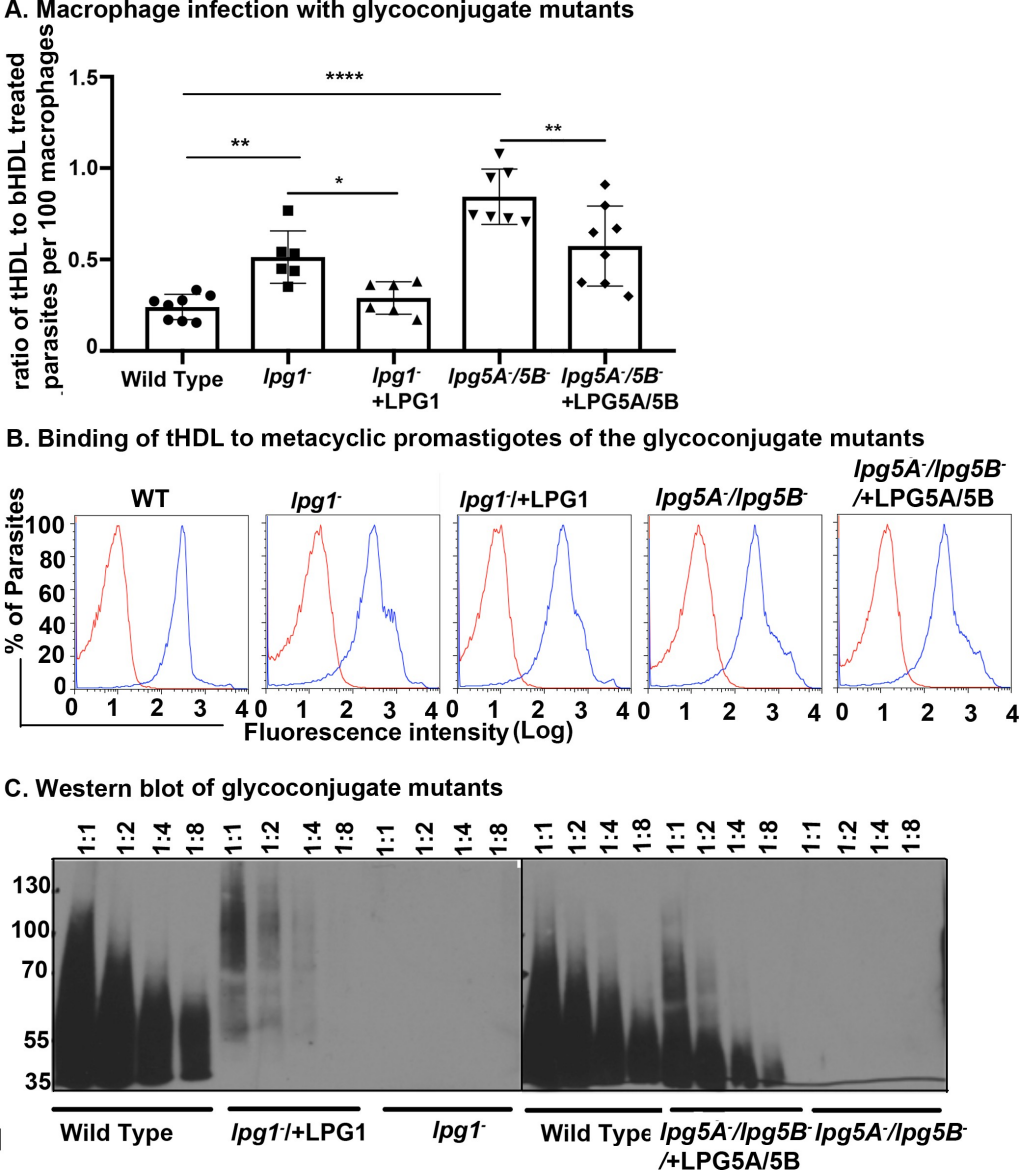
*S*urface glycoconjugate mutants of *L*. *major* are less susceptible to TLF-mediated lysis than wild-type parasites in the macrophage. Metacyclic promastigotes of *L*. *major* (WT), the glycoconjugate mutants (*lpg1^-^* and *lpg5A*^*-*^ */lpg5B*^*-*^), or the complemented lines (*lpg1^-^*+LPG1 and *lpg5A*^*-*^*/lpg5B*^*-*^+LPG5A/5B) were: **A.** used to infect macrophages in the presence of human HDL(tHDL) or bovine HDL (bHDL) (1.5 mg/ml). Parasites were counted in macrophages at 24 hours post-infection by staining with DAPI. The y-axis represents the ratio of parasites per 100 macrophages resulting from tHDL treatment compared to bHDL treatment. The data are representative of three experiments combined, * *p*< 0.05, ** *p*<0.001, and **** *p*<0.0001. ANOVA test, multiple comparisons with Bonferroni correction. **B.** co-incubated with 10 μg/ml of DyLight-488 labelled tHDL (blue) at 4°C for 30 min. The fluorescence was measured using flow cytometry where untreated parasites (red) were used as control. **C.** were lysed (2x10^7^) and the extracts were then serially diluted to compare the expression of phosphoglycan (PG) proteins, which were probed using anti-phosphoglycan antibody WIC 79.3.

Amastigotes, which are the pathogenic forms of *Leishmania*, have distinct surface glycoconjugate components compared to the infective metacyclic promastigote stage [23]. Therefore, we hypothesized that surface glycoconjugates attached to the plasma membrane of the parasite play an important role in TLF-binding and hence TLF-susceptibility. To directly evaluate the role of the surface glycoconjugates in the susceptibility to TLF-mediated lysis of metacyclic promastigotes, we utilized mutant strains of *L*. *major* parasites deficient in the synthesis of specific surface structures. The *lpg1*^-^ mutants lack the lipophosphoglycan (LPG) core galactofuranosyl transferase LPG1 and are specifically deficient in LPG synthesis [27]. The *lpg5A*^*-*^*/lpg5B*^*-*^ double mutants cannot import UDP-Galactose (UDP-Gal) into the Golgi and are therefore defective in the synthesis of multiple glycoconjugates including LPG and proteophosphoglycan (PPG) [28]. We tested the protective effect of TLF against these mutants by infecting macrophages with *lpg1*^-^ or *lpg5A*^*-*^*/lpg5B*^*-*^ metacyclic promastigotes in the presence of tHDL or bHDL. Wild-type *L*. *major* parasites were used as a control. The metacyclic promastigotes of *lpg1*^*-*^ and *lpg5A*^*-*^*/lpg5B*^*-*^ mutants were less susceptible to TLF-mediated lysis compared to the wild-type *L*. *major* strain (Fig 5A).

The *lpg5A*^*-*^*/lpg5B*^*-*^ mutants, lacking both LPG and PPG, were more resistant to TLF-mediated killing compared to the *lpg1*^*-*^ mutants devoid of LPG only. To test if the resistance of these metacyclic promastigote mutants to TLF-mediated killing is due to the inability of tHDL to bind to the parasites, we investigated the binding of labelled tHDL to the metacyclic promastigotes of the mutant parasites. We found that both *lpg1*^*-*^ and *lpg5A*^*-*^ */lpg5B*^*-*^ mutants were still able to bind labelled tHDL (Fig 5B) suggesting that the loss of TLF lysis is not due to inability of the tHDL to bind to the parasites. Complemented cells expressing *LPG1* (Fig 4A) demonstrate WT phenotypes but tHDL (TLF) susceptibility was only partially restored in the complemented line *lpg5A*^*-*^*/lpg5B*^*-*^*/+LPG5A+LPG5B* (Fig 5A). To confirm that the complemented lines were producing glycoconjugates, we screened the level of phosphoglycans in these mutants and compared to the wild-type *L*. *major*. Although the level of phosphoglycans was not restored to the wild-type parasites’ level, the complemented lines produce phosphoglycans (Fig 5C). Accordingly, the susceptibility of the parasites to TLF were partially restored in the complemented lines (Fig 5A and 5C). Our results support the hypothesis that the surface glycoconjugates of metacyclic promastigotes contribute to the susceptibility of TLF-mediated lysis in macrophages.

### The resistance of surface glycoconjugate mutants to TLF activity is not due to variations in phagosomal pH

**Fig 6:**
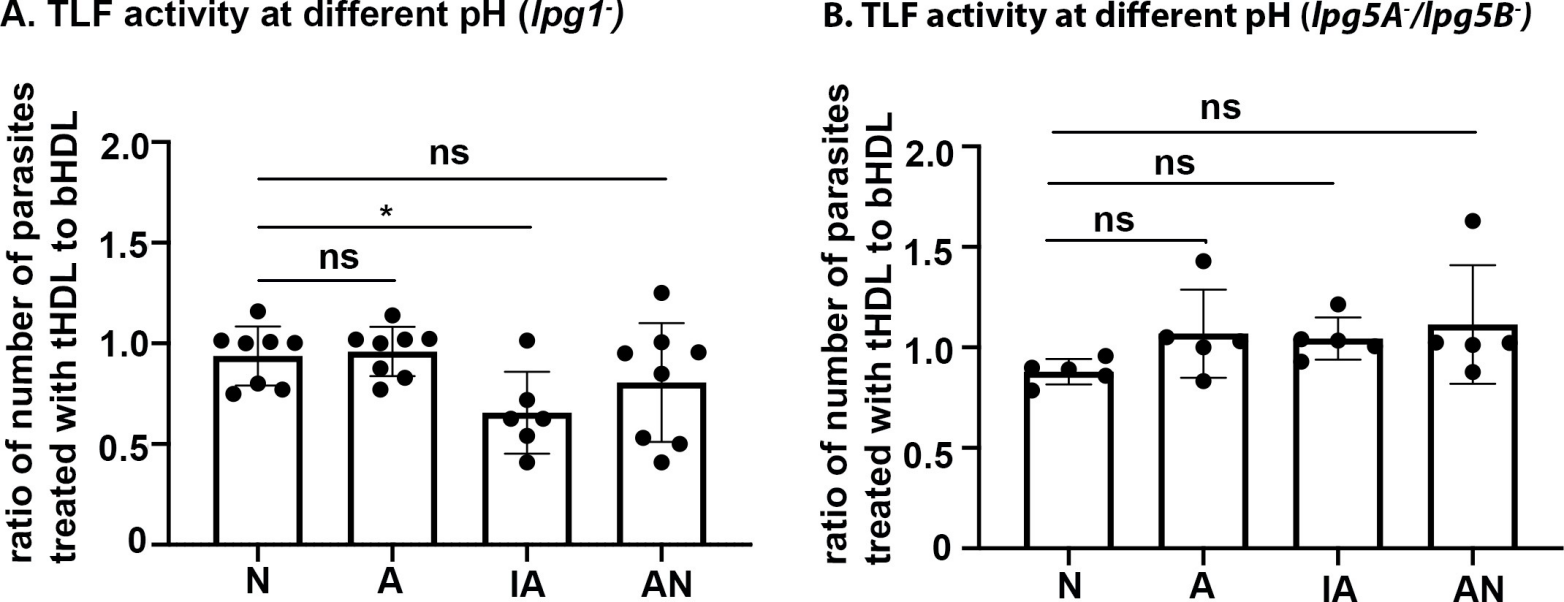
Resistance of surface glycoconjugate mutants to TLF activity is not due to variations in phagosomal pH. Metacyclic promastigotes of the glycoconjugate mutants A. *lpg1^-^* and B. *lpg5A*^*-*^ */lpg5B*^*-*^ were treated with human HDL (tHDL, 1.5 mg/ml) or bovine HDL (bHDL, 1.5 mg/ml) and incubated in Neutral—N pH 7.4 for 2hours, Acidic–A pH 5.6 for 2 hours, Increased acidification- IA pH 5.6 for 1 hour followed by pH 4.5 for 1 hour, and Acid and Neutral- AN pH 5.6 for 1 hour followed by pH 7.4 for 1 hour. Parasites were microscopically counted using a hemocytometer. The y-axis represents the ratio of parasites resulting from tHDL treatment compared to bHDL treatment. The data represents the Mean ± standard deviation (SD) of three experiments that have been combined. * *p*<0.05, ns- non-significant, ANOVA test, multiple comparison with Bonferroni correction.

Metacyclic promastigotes of *Leishmania* sp. inhibit the biogenesis and maturation of macrophage phagosome and shed surface phosphoglycan glycoconjugates within 1–2 hours of infection [21,22,24]. Mutant parasites deficient in glycoconjugates are consequently impaired in phagosomal maturation. Therefore, we tested if the resistance of metacyclic promastigotes to TLF-mediated lysis in the glycoconjugate mutants could be associated with alterations in the phagolysosomal pH of macrophages infected with the glycoconjugate mutant parasites by again employing *in vitro* phagosome-like pH conditions. LPG deficient parasites were not lysed by TLF in neutral (N), acidic (A), or acid and then neutral (AN) conditions (Fig 6A). There was a significant TLF-mediated reduction in parasite number upon increasing acidification (Fig 6A). However, this reduction was less than that observed for the wildtype parasites in the same treatment condition (IA) (Fig 4A), suggesting that upon shedding LPG, metacyclic promastigotes become resistant to TLF-mediated lysis. The *lpg5A*^*-*^*/lpg5B*^*-*^ mutants were resistant to TLF-mediated lysis in all pH conditions indicating that *lpg5A*^*-*^*/lpg5B*^*-*^ mutants are more resistant to TLF activity than *lpg1*^*-*^ supporting the macrophage infection results and highlighting potential roles for both LPG and PPG in TLF-susceptibility.

## Discussion

TLF is an innate immune complex in humans and some non-human primates that was originally discovered due to its protective effect against African trypanosomes. Previously, we have shown that human TLF also ameliorates the infection caused by intracellular kinetoplastid parasites, *L*. *major* and *L*. *amazonensis*, both in macrophages and in a subcutaneous model of infection in transient transgenic mice [8]. In the subcutaneous model, macrophages are the predominant cell that phagocytose the parasites early in the infection [3]. Therefore, it can be inferred that TLF lyses metacyclic promastigotes of *L*. *major* in macrophages *in vivo*. However, the role that TLF plays during natural intradermal infections by *Leishmania* is not known. In this study, we found that TLF can ameliorate infection by cutaneous *L*. *major in vivo* when mice are infected intradermally with metacyclic promastigotes (Fig 1). The TLF proteins are detectable in plasma for 3 days post infection suggesting that TLF kills the metacyclic promastigotes within the first 3 days of infection. Therefore, TLF-mediated innate immunity could be important during natural sand fly infections in humans.

TLF activity has been detected in humans, gorillas, baboons, mandrills, and sooty mangabeys [9,10,29]. While hominoid (human and gorilla) TLF only protects against animal-infective trypanosomes, old-world monkeys (OWMs) have TLFs that confer resistance against both human and animal-infective trypanosomes. This differential activity is attributed to differences in the APOL1 sequence which is approximately 60% identical between humans and baboons [9]. Here, we found that both human and baboon TLFs protect against *L*. *major* (Figs 1 and 2). However, transiently-transgenic mice (generated by HGD) producing human TLF were able to restrict *Leishmania* growth higher than germline-targeted transgenic mice producing baboon TLF (Figs 1 and 2B). This difference could be due to: 1) A mechanistic difference in activity between human and baboon TLF; 2)an increase in recruited immune cells induced by HGD (S1B Fig); or 3) differences in the concentrations of circulating TLF (S2A Fig) produced by the two methods. Although all three remain a possibility that need to be tested, our data provides strong evidence to support that the differential killing of *L*. *major* observed in transiently transgenic mice and germline transgenic mice is due to the difference in the level of circulating TLF in these mice (Figs 1, 2 and S2A). The homozygous germline transgenic mice produce twice as much TLF-associated protein (APOL1- 1:40 dilution; HPR 1:160 dilution) compared to heterozygous mice (APOL1- 1:20 dilution; HPR- 1:80 dilution) (S2C Fig). As expected, homozygous germline transgenic mice show a 10-fold reduction in parasite burden (p = 0.0031) compared to a 5-fold reduction in heterozygous mice (p = 0.2464) (Fig 2C). In addition, we found that the TLF-mediated reduction in the parasite burden in the mice was also dependent on the initial infective dose (Fig 2). In a natural infection, the disease outcome possibly depends on the number of parasites that the sand fly injects as well as the circulating level of TLFs in each individual. Therefore, low HDL level at the time of infection could conceivably exacerbate *L*. *major* infection. In fact, symptomatic infection by visceral *Leishmania* strains has been associated with a reduction in HDL compared to the healthy control subjects in human studies [30].

Following infection, *L*. *major* recruits phagocytic immune cells to the site of infection [2,3,26]. Although the number and type of these phagocytes are variable depending on the route of infection [3], neutrophils represent one of the predominant cells that phagocytose the parasites early post-infection irrespective of the infective route [3,26]. Neutrophil depletion experiments in mice have shown discrepant results in terms of whether the role of neutrophils is primarily protective or deleterious for *Leishmania* [2,3,5,21,31–33]. Notably, these early experiments were performed in wild-type mice that do not produce TLF and we were able to corroborate these results ourselves (Fig 3C). In contrast, we observed a significant increase in the parasite burden in TLF-producing mice depleted of neutrophils compared to the wild type mice with neutrophils (Fig 3C). Furthermore, there were fewer parasites in the presence of tHDL compared to bHDL in *ex vivo* infection of bone marrow isolated neutrophils (Fig 3D). This TLF mediated lysis of parasites in *ex vivo* neutrophil infection was apparent only in the presence of TLF enriched human HDL but not in human HDL collected after density gradient centrifugation that was not enriched in TLF (panels i and ii in S4B Fig). Noteworthy, we found that presence of endotoxin in the HDL preparation drastically increased the parasite count in the neutrophils (panel iii in S4B Fig). It is therefore important to make sure to use endotoxin free HDL for such assays. These results support our hypothesis that TLF kills *L*. *major* metacyclic promastigotes within neutrophils and opens the possibility that TLF can act on *L*. *major* within the acidic phagosomes of other immune cells, which should be investigated in future studies.

The *Leishmania* surface glycoconjugate LPG neutralizes the phagosomal pH after 2 hours of infection and prevents any re-acidification thereafter [4,20,22,24]. Therefore, TLF activity against parasites within acidic phagosomes of immune cells must occur within the first 1–2 hours post-infection, while the vacuole is still acidic. The killing of metacyclic promastigotes within the first few hours of infection are especially critical for short-lived cells like neutrophils with half-lives of 7–9 hours. Previously, we demonstrated that TLF can kill *L*. *major* metacyclic promastigotes at acidic pH when incubated for 24 hours [8]. Here we analyzed the susceptibility of TLF against metacyclic promastigotes at various pH conditions in short term killing assays *in vitro* (2hr). We observed 100% lysis when parasites were subjected to increasing acidification (IA) from pH 5.6 to pH 4.5 in the presence of tHDL, mimicking naturally maturing phagosomes (Fig 4A). The APOL1 channels are pH gated and require neutralization for opening to allow ions to flow. Therefore, we do not know the mechanism of lysis in metacyclic promastigotes in the “increasing acidification” condition. We attempted to investigate this using artificial bilayers using recombinant APOL1. However, artificial bilayers were unstable below pH 5 in the presence of APOL1. We interpret these data as the possible increase in insertion of APOL1 into membrane bilayer at acidic pH that leads to membrane disruption.

There is partial but significant lysis of parasites upon acidification at pH 5.6 followed by neutralization (AN), mimicking change in phagosomal pH in the presence of phosphoglycan such as LPG (Fig 4A) [4,20–22,24] and allowing for neutral-pH-triggered opening of APOL1 ion channels. Nonetheless, there are always some parasites that survive and establish an infection in the presence of TLF in both *ex vivo* macrophage infections and *in vivo* mouse infections (Figs 1, 2 and 5A). Therefore, it is highly likely that infected immune cell phagosomes follow the acidification and then neutralization route and not the gradual acidification that leads to 100% lysis of parasites. This is analogous to the APOL1-mediated lysis model as observed in artificial bilayers, where recombinant APOL1 inserts into the bilayer and forms a closed-channel at acidic pH, although still permitting slow movement of ions in artificial bilayers [13,17]. Accordingly, incubating metacyclic promastigotes with tHDL at pH 5.6 (A) for 1–2 hours is not sufficient to lyse the parasites (Fig 4A). However, if incubated for a long time (24 hours), TLF could lead to swelling and lysis of the metacyclic promastigotes due to slow leakage of ions through the closed channel [8]. This closed channel subsequently opens upon neutralization allowing more robust movement of cations [17]. We hypothesize that the APOL1 protein of the TLF complex forms cation channels in the parasite plasma membrane that eventually leads to membrane depolarization and lysis, although this hypothesis requires further investigation.

There is a fundamental difference in the mechanism of lysis of *Leishmania* by TLF compared to African trypanosomes. In African trypanosomes, when TLF or APOL1 is co-incubated with parasites at neutral pH (blood and extracellular fluid), the parasites endocytose TLF/APOL1, which inserts into the membrane of acidic endo/lysosomes to form a closed ion channel. The pH-gated ion channel opens upon neutralization, which occurs when the endo/lysosomal membrane is recycled to the parasite plasma membrane leading to ionic imbalance, osmotic influx of water and lysis of the parasites [13,17,34,35]. In contrast, incubation of tHDL with *L*. *major* metacyclic promastigotes in a neutral pH does not kill the parasites (Fig 4A) and [8]. Furthermore, we have been unable to obtain any evidence suggesting that *Leishmania* parasites endocytose TLF. However, increasing acidification (pH 5.6 to pH 4.5) or acidification followed by neutralization (pH 5.6 to pH 7) in the presence of tHDL results in parasite lysis (Fig 4A). Therefore, the lysis of *L*. *major* metacyclic promastigotes by TLF requires an external acidic pH, such as that which is present within the phagosomes of immune cells, allowing APOL1 to form ion channels in the surface of the parasites. This is further supported by the fact that amastigotes with a different glycoconjugate composition compared to metacyclic promastigotes, do not bind TLF and are resistant to TLF-mediated lysis (Fig 4B and 4C). Moreover, mutant parasites deficient in surface glycoconjugates are also resistant to TLF-mediated lysis. These data provide strong evidence to support the hypothesis that TLF binds to the surface of metacyclic promastigotes, allowing APOL1 to form pH-gated ion channels in their plasma membrane leading to their colloidal osmotic lysis. Neutrophil recruitment early in the infection is essential for TLF activity *in vivo*, *suggesting* that TLF kills the metacyclic promastigotes of *L*. *major* within neutrophils.

**Fig 7:**
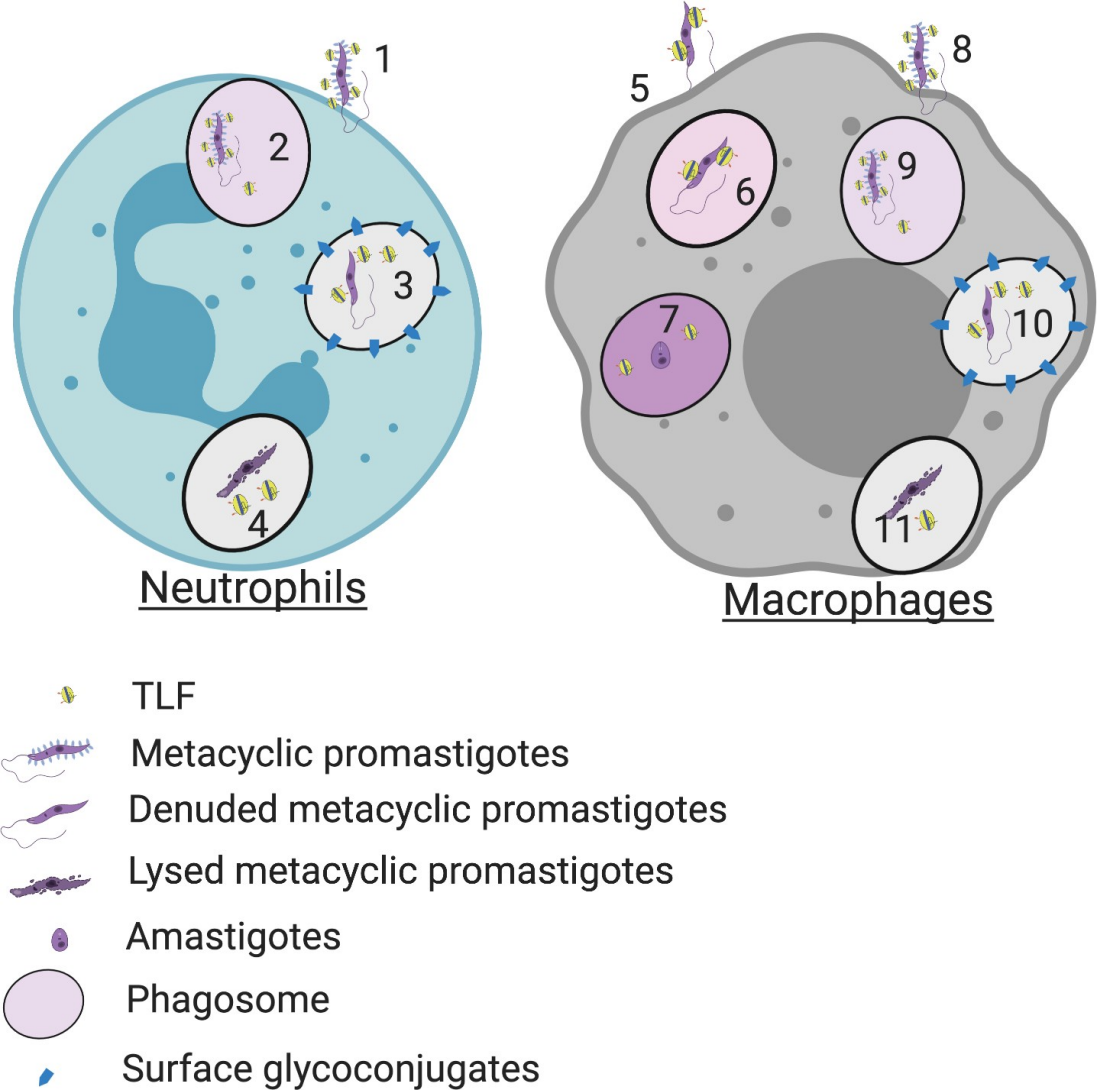
Proposed model of TLF activity against *L*. *major*. 1. TLF interacts with metacyclic promastigotes of *L*. *major* and TLF-bound metacyclic promastigotes predominantly infect neutrophils in the early hours of infection 2. Within the acidic pH of immune-cell phagosomes, TLF, the APOL1 ion channel is primed. 3. Metacyclic promastigotes shed their surface glycoconjugates (blue arrow heads), which inhibit phagosomal maturation and neutralize the pH of phagosomes. 4. TLF damages and lyses (due to open APOL1 cation channels in the parasite plasma membrane) some metacyclic promastigotes of *L*. *major*. 5. Some metacyclic promastigotes that escape TLF lysis are released into the extracellular milieu that then infect other immune cells like macrophages. 6. TLF-bound denuded metacyclic promastigotes are resistant to TLF lysis. 7. In the macrophage phagolysosomes, metacyclic promastigotes transform into amastigotes. Amastigotes are resistant to TLF and grow and divide in macrophages. 8. TLF bound metacyclic promastigotes can infect macrophages. 9. In acidic phagosomal pH, TLF, the APOL1 ion channel is primed. 10. Metacyclic promastigotes shed their surface glycoconjugates (blue arrow heads), which inhibit phagosomal maturation and neutralize the pH of phagosomes. 11. Metacyclic promastigotes are lysed in the macrophage phagosomes, by TLF due to open cation channels in the parasite plasma membrane. Illustration created with BioRender.com.

Denuding parasites of surface glycoconjugates of *Leishmania* sp. renders them resistant to TLF-mediated lysis (Fig 5A) suggesting that TLF interacts with the surface glycoconjugates of the metacyclic promastigotes. Metacyclic promastigotes shed their surface glycoconjugate LPG into the phagosome membranes, which interferes with phagosomal maturation [4,20–22,24]. This shedding of phosphoglycans can happen when the parasites infect neutrophils [4]. Therefore, APOL1 may insert into the parasite plasma membrane at a slightly acidic pH and then experience neutral pH when the parasites shed their glycoconjugates to interfere with the phagosomal pH in the immune cells such as neutrophils. Short-lived apoptotic neutrophils then release the metacyclic promastigotes that go on to infect other immune cells like macrophages [2]. It is likely that metacyclic promastigotes released from neutrophils are therefore denuded to various degrees of their surface phosphoglycan. Therefore, we tested the effect of TLF on the denuded metacyclic promastigotes by utilizing mutant parasites (*lpg1*^*-*^ and *lpg5A*^*-*^/5B^-^) deficient in the synthesis of surface glycoconjugates. Our binding experiments show that tHDL binds to surface glycoconjugate deficient parasites similarly to wild-type metacyclic promastigotes (Fig 5B). However, this binding of tHDL to the glycoconjugate deficient metacyclic promastigotes is not sufficient to lyse the parasites (Fig 5A). In fact, bHDL, which does not kill parasites, also shows binding to the metacyclic promastigotes of *Leishmania* (S5 Fig), suggesting that additional interactions between TLF and surface glycoconjugates are required for TLF-susceptibility. The fluorescence intensity of tHDL bound parasites (mfi difference of tHDL bound parasites and autofluorescence of 268.19) is greater than the bHDL (mfi difference of bHDL bound parasites and autofluorescence of 2.8) parasites, which is possibly due to the difference in labelling of the HDL by Dylight-488. In our hands, labelling of bHDL was always less efficient than tHDL possibly due to the difference in the protein composition between two complexes. For example, the TLF complexes are characterized by presence of 60% proteins, 40% lipids (density- 1.21–1.24) compared to other HDL complexes 50% proteins, 50% lipid (density- 1.063–1.21 g/ml) [35]. Our results show that parasites deficient in the synthesis of LPG due to mutation in *lpg1*^*-*^ are slightly resistant to TLF activity compared to the wild type metacyclic promastigotes (Figs 5A and 6A). However, *lpg5A*^*-*^ */lpg5B* with deficiency in UDP-galactose transporter and hence a more severe effect on surface phosphoglycans are completely resistant to TLF activity (Figs 5A and 6B). These results suggest that TLF mediated lysis of metacyclic promastigotes requires surface glycoconjugates on the parasites. Therefore, shedding of glycoconjugates is advantageous for the parasites in evading the TLF mediated lysis that would eventually lead the parasites to transition to TLF-resistant amastigotes (Fig 7). Virulent parasites such as *L*. *mexicana* has surface glycoconjugates in amastigote stages while the metacyclic promastigotes do not have glycoconjugates such as glycoinositol phospholipids [36]. Whether TLF provides innate immunity against these parasites should be studied in the future.

Our results show that TLF has evolved in primates as an innate immune factor that kills extracellular African Trypanosomes and intracellular *Leishmania* sp.. The parasites have evolved to overcome the TLF activity and hence can eventually survive, divide, and proliferate to cause disease pathogenesis. The African Trypanosome species *Trypanosoma brucei rhodesiense and T*. *b*. *gambiense* have evolved to partially evade TLF activity and cause human African trypanosomiasis [29]. Likewise, *Leishmania* sp. have evolved to transition into amastigotes with different surface glycoconjugates that can eventually grow in the host immune cells to manifest disease. This transformation of parasites into amastigotes form is advantageous for the parasites to evade host innate immunity such as TLF lysis. Our data suggest that innate immunity afforded by TLF may be effective against a broader range of intracellular pathogens coated with diverse glycoconjugate moieties.

## Materials and methods

### Ethics statement

All animal experiments were approved by the Hunter College Institutional Animal Care and Use Committee (IACUC); animal welfare assurance agreement number D16-00413 (A3705-01) from the National Institute of Health, office of Laboratory animal welfare.

### Parasites and media

*L*. *major* strain Friedlin V1 (MHOM/JL/80/Friedlin) or *L*. *amazonensis* (IFLA/BR/67/PH8), *lpg1*^*-/-*^, *lpg1*^*-/-*^/*+LPG1*, *lpg5A*^*-*^*/lpg5B*^*-*^*/+LPG5A+LPG5B* promastigotes were grown in M199 medium (Gibco) [27,28]. The parasites were grown to stationary phase culture. Infective-stage metacyclic promastigotes were isolated by density centrifugation on a Ficoll (Sigma, F8016) gradient from a 8–10 day old culture [37]. Axenic amastigotes were cultured by taking the stationary phase of the promastigote culture and acidifying to pH 5.5 followed by incubation at 33 degrees.

### Murine transfection and infection

Transiently transgenic mice were created by intravenously injecting 50 μg of plasmid DNA (*APOL1*:*HPR*) by hydrodynamic gene delivery (HGD) as described previously [38]. One day post-HGD, plasma was collected by a tail bleed from a small drop of blood. Bleeding was stopped with Kwik-Stop Stypic antiseptic. Plasma levels of the TLF proteins were analyzed by western blot using a rabbit polyclonal anti-APOL1 antibody (ProteinTech 16139-1-AP—1:10,000) or a rabbit polyclonal anti-HP antibody (Sigma H8636–1:10,000) as the primary antibody and TrueBlot anti-rabbit secondary antibody (Rockland Antibodies 18-8816-33–1:5000). Transiently transfected mice were intradermally infected on the same day (1 day post-HGD transfection) with 10^4^ Ficoll (Sigma) purified metacyclic promastigotes in the ear. This dose was chosen based on previous studies showing that a sand fly injects a wide range of parasites intradermally during its blood meal that ranges from 10–10^5^ metacyclic promastigotes per bite [25]. The sand fly infection dose follows a bimodal distribution with a high median dose ranging from 10^3^–10^4^ parasites and the lower median dose from 100–600 parasites [25]. Parasite doses ranging from 100 to 10^4^ were intradermally injected into mice as indicated in the figures. For parasite quantification, ears were harvested on day 15 post-infection and quantified by qPCR as described below.

Germline transgenic mice were generated by targeted recombination of *APOL1* and *HPR* into the *ROSA26* locus. The baboon *APOL1* gene is under the control of a ubiquitin promoter and the baboon *HPR* gene is under the control of an albumin promoter. The *ROSA26* locus is used in mice for constitutive expression of genes. The mice were made by Regeneron Pharmaceuticals Inc. and transferred by an MTA to City University of New York Research Foundation. *APOL1* is expressed in various tissues in primates [37]. Expression of *HPR* is however, limited to the liver [38]. Our germline murine model thus resembles the expression of these genes in the primates.

### APOL1 and HPR produced in plasma from germline transgenic mice

Plasma was collected by tail bleed from germline transgenic homozygous and heterozygous mice expressing dual genes *APOL1* and *HPR* to produce APOL1 and HPR proteins of the TLF complex. For comparing APOL1 and HPR production, the plasma samples were serially diluted and separated by SDS PAGE gel (non-reducing) as described above. The proteins were then probed for baboon APOL1 using anti-baboon APOL1 antibody (rabbit anti-sera raised against the following peptide: CSVEERARVVEMERVAESRTTEVIRGAKIVDK, AnaSpec Incorporated, 1:1000) and anti-Hp antibody (H8636–1:10,000). Baboon HPR has two predicted glycosylation sites and hence runs higher (~ 55 kDa) than its expected size of 42 kDa [9].

### Parasite number determination by qPCR

Ears were harvested 15 days post-infection after euthanizing the mice. Whole ears were cut and washed in ethanol for 5 minutes followed by digestion in trizol (ZYMO Research). DNA was isolated from ears using the DNeasy Blood and Tissue Kit (Qiagen, 69506). Parasite DNA was quantified using previously described primers that amplify the kinetoplastid DNA (kDNA): forward– 5’- CCTATTTTACACCAACCCCCAGT-3’ (JW11); reverse—5’-GGGTAGGGGCGTTC TGCGAAA-3’ (JW12) [39]. The murine housekeeping genes used were GAPDH amplified using previously described primers: GAPDH-F 5’- TTTGATGTTAGTGGGGTCTCG-3’; GAPDH-R 5’-AGCTTGTCATCAACGGGAAG-3’ [40,41]. PCR was performed using the Applied Biosystems ViiA Real Time PCR System in a 20 μl reaction mix with 10 μl SYBR Green master mix (Applied Biosystems), 1 μmol each of the forward and reverse primers and 50ng of the template. The PCR was run for 40 cycles at 95°C for 10 sec, 60°C for 15 sec and 72°C for 30 sec. Parasite Ct were normalized against mouse GAPDH Ct and the numbers of parasites were quantified using the standard curve of mouse ear tissues with known numbers of *Leishmania* parasites (limit of linear detection ranges from 10^2^ to 10^6^ parasites per ear).

### Neutrophil depletion and infection

Neutrophils were depleted from targeted transgenic mice expressing baboon TLF (APOL1 and HPR homozygotes) and wild-type C57BL/6NTac (Taconic) mice not expressing TLF by injecting anti-mouse Ly6G [31], clone 1A8 antibodies intraperitoneally (BioXcell, BP0075-1, 1 mg). Isotype matched IgG2a, clone 2A3 (BioXcell, BP0089, 1mg) antibody was injected as a control. Blood was collected via tail bleeding 24 h later and stained with PE Rat Anti-Mouse CD11b- clone M1/70, FITC Rat Anti-Mouse Ly-6G and Ly-6C clone RB6-8C5, APC Rat Anti-Mouse Ly-6C- Clone AL-21 (BD Pharmingen) for 30 minutes in the dark. The samples were then treated to BD FACS lysing solution for 10 min at room temperature followed by two washes. Flow cytometry was performed with a Becton Dickinson FACSCalibur system and Becton Dickinson FACScan.

After blood collection mice were infected with metacyclic promastigotes (100 for ear infection kinetics, and Giemsa, and 10^6^ for neutrophil analysis in ears by FACS). To check neutrophil recruitment, ears were harvested from mice 12 hours post-infection (peak neutrophil recruitment time) [26] and analyzed by flow cytometry or 10 hours post-infection and imprinted onto slides followed by methanol fixation and stained with Giemsa. To check by flow cytometry, the ears were digested with 0.2mg/ml Liberase (Roche; 05401020001) for 2 hours at 37°C. Cells were placed on ice and stained as described above for blood and assayed by flow cytometry.

### Purification of human HDL, bovine HDL and mouse HDL

The HDL from normal human plasma was purified by density gradient centrifugation as described elsewhere [18,42]. The plasma density was adjusted to 1.25 g/ml using potassium bromide and centrifuged at 49,000 rpm (NVTi 65; Beckman) for 16 hours at 10°C. The top lipoprotein fraction was collected (total lipoprotein) followed by density adjustment to 1.3 g/ml by adding additional potassium bromide. Then 4 ml of the adjusted lipoprotein was layered under 8 ml of 0.9% NaCl and centrifuged at 49,000 rpm for 4 hours at 10°C (NVTi 65 rotor; Beckman). The HDL (lower band) was harvested and dialyzed in Tris-buffered saline [TBS; 50 mM Tris-HCl, 150 mM NaCl (pH 7.5)] at 4°C and concentrated by ultrafiltration (Amicon Ultra-15 Centrifugal Filter Units, MWCO 100 kDa). This concentrated HDL is called (hfHDL), which was further purified based on the difference in their molecular size by size exclusion chromatography using a Superose 6 column (1.5x60cm) (GE Healthcare) via Fast Protein Liquid chromatography (V_0_ = 34ml, flow rate = 0.4ml/min, fraction size = 1.5ml). The TLF enriched HDL fractions that showed 50% or higher trypanolysis were collected and concentrated [11]. This TLF enriched human HDL is called tHDL.

We found it important to use tHDL for our assays. Human HDL not enriched in TLF collected after density gradient centrifugation (hfHDL) had same number of parasites as bovine HDL collected after density gradient centrifugation (bfHDL) emphasizing the role of TLF in reducing parasite burden (panels i and ii in S4B Fig). In addition, any presence of endotoxin in the tHDL changed the dynamics of parasite number in the neutrophils at 4 hours post infection (panel iii in S4B Fig) which was compared to bovine HDL collected after density gradient centrifugation (bfHDL). From these results we found that it is important to compare HDL collected in similar way i.e. tHDL and bHDL and is free from endotoxins, which we used for all of our *in vitro* assays. We also observed a reduction in TLF activity when the HDL samples were freeze-thawed (panel ii in S4B Fig). Therefore, we aliquoted all of our isolated tHDL enough for one experiment to avoid freeze-thaw of the samples.

The predominant lipoprotein fraction in bovine is high-density lipoproteins. Therefore, for the bovine HDL separation, we did a density gradient centrifugation at 1.25 g/ml. The top band of the lipoprotein was collected and concentrated. For neutrophil experiments, a second density gradient centrifugation was performed as described for human HDL. The HDL band was collected and concentrated as described above which is called bfHDL. The bfHDL was further purified by size fractionation on a Superose 6 column (GE Healthcare) equilibrated with TBS. Only the fractions containing APOA1, the structural protein of HDL were then pooled, concentrated, and aliquoted and is called bHDL.

For mouse lipoprotein purification, plasma density was adjusted to 1.25 g/ml and lipoproteins (top fraction) were collected by density gradient centrifugation as described above. The potassium bromide was then dialyzed out. When separating the plasma lipoproteins by size exclusion chromatography directly, 100 μl of mouse plasma was separated using a superdex 200 10/300 column, (GE Healthcare) (V_0_ = 24ml, flow rate = 0.5ml/min, fraction size = 1.0ml). Lipoprotein fractions were analyzed for the presence of the TLF proteins by western blot as described above.

### Test HDL for the presence of endotoxins

The isolated HDL samples were diluted in sterile endotoxin-free water (1:50 dilution) and then tested for the presence of endotoxins with Pyrotell Gel-Clot Formulation (Associates of Cape Cod, Inc) following the manufacturer’s protocol.

### Infection of neutrophils

Bone marrow derived neutrophils were isolated from C57/B6 mice using EasySep Mouse Neutrophil Enrichment Kit (STEMCELL, 19762) following manufacturer protocol. Metacyclic promastigotes were labelled with Carboxyfluorescein succinimidyl ester (CFSE) by incubating for 15 minutes at room temperature followed by wash. Isolated neutrophils were infected with CFSE stained metacyclic promastigotes at multiplicity of infection (MOI) of 3 metacyclic promastigotes per neutrophil. The infection was performed in the presence of tHDL and bHDL for four hours. After 4 hours, infected neutrophils were stained with Live-dead Fixable far red (ThermoFisher, L10120) and Ly6-G PE (BD Biosciences, 551461), fixed with PFA and assayed by flowcytometry.

### Infection of macrophages

Bone marrow derived macrophages (BMDM) were isolated from BALB/c mice and cultivated in DMEM + 15% v/v L cell supernatant as described previously [43]. 7 day old BMDM were replated at a density of 50,000 cells per well in 48 well plates with coverslips in DMEM (Corning Cellgro). *L*. *major* metacyclic promastigotes were opsonized for 30 min in DMEM containing 4% A/J mouse serum. BMDM were infected at a MOI of 3 metacyclic promastigotes per macrophage for 2 hours at 33°C in the presence of tHDL or bHDL (5% CO_2_, 95% air humidity). Thereafter, non-phagocytosed parasites were washed off with prewarmed DMEM and the media was replaced with fresh media with HDL (1.5 mg/ml tHDL and bHDL). The cultures were further incubated for 24 hours. Intracellular parasites were quantified after staining with DAPI (3 μmol/L) by a Leica TCS SP2 AOBS confocal laser-scanning microscope.

### *Leishmania in vitro* assay

Human HDL or bovine HDL (1.5 mg/ml, physiological concentration in blood) was added to parasites in DMEM with 0.2% Bovine Serum Albumin (BSA, Sigma) (Ficoll purified metacyclic promastigotes or axenic amastigotes) at neutral pH 7 (mimics the extracellular environment) followed by acidification to pH 5.6 using succinate buffer (0.05M succinic acid and 0.015M NaOH, pH 3.8) to mimic the lumen of the phagosome. Parasites were incubated at 33°C (5% CO_2_, 95% air humidity) for 1 hour. Parasites with HDL were then subjected to a second acidification to pH 4.5, (Increasing acidification, IA) or neutralization (Acid then Neutral, AN) to pH 7 and incubated for another hour at 33°C (5% CO_2_, 95% air humidity). To assess the number of remaining intact parasites, we used a hemocytometer.

### Parasite centrifugation and washes

Purified *L*. *amazonensis* metacyclic promastigotes or axenic amastigotes (1 x 10^6^/ml) were centrifuged (4°C, 8000 x g, 1 min) to obtain the pellet. For subsequent washes, parasites were resuspended in DMEM, 0.2% BSA and centrifuged as described above.

### HDL labeling and binding to parasites

tHDL and bHDL were labeled with DyLight 488 NHS ester (ThermoFisher, 46402) according to the manufacturer’s instructions. Purified *L*. *amazonensis* metacyclic promastigotes or axenic amastigotes (1 x 10^6^/ml) were washed twice at 4°C as described earlier and incubated with 10 μg/ml Dylight-488 labeled tHDL/bHDL in DMEM, 1% BSA for 30 min on ice. Cells were washed twice at 4°C as described above before being analyzed by flow cytometry. Flow cytometry was performed with a Becton Dickinson FACSCalibur system.

### Detection of surface glycoconjugates

To test for the presence of surface glycoconjugates, 2x10^7^ metacyclic promastigotes of wild-type, complemented lines (l*pg1*^*-/-*^+LPG1, l*pg5A*^*-*^*/lpg5B*^*-*^*/*+LPG5A/5B) and mutants (l*pg1*^*-/-*^ l*pg5A*^*-*^*/lpg5B*^*-*^) were lysed in 1% NP-40 in the presence of an protease inhibitor cocktail (Sigma, 11836170001). Equal cell equivalents were separated on a 10% SDS PAGE (Biorad, 4561036) gel followed by western blot using anti-phosphoglycan antibody WIC79.3 (1:1000; gift from Dr. Steve Beverley, WU), followed by anti-mouse (1:10,000, Proteintech, SA00001-1) secondary antibody and detected by chemiluminescence.

### Statistical analysis

Statistical analysis was performed as described in figure legends using Prism software for Mann-Whitney U test and ANOVA with Bonferroni correction. For Fig 3, Bartlett test, Shapiro-Wilk Normality test, Tukey test and Kruskal-Wallis test were performed in R [44] using the package WSR2 [45].

## Supporting information

S1 Fig Production of the TLF proteins (APOL1 and HPR) and the effect of HGD transfection on the myeloid cell count in the blood. Mice were subjected to HGD and blood was collected **A**. Plasma from mice collected on 1, 2 and 3 days post HGD were serially diluted and separated by SDS PAGE followed by immunoblotting using anti-APOL1 (for APOL1) and anti-HP (for HPR) antibodies, *HP- Human Plasma. **B**. Blood was collected 24 hours post-HGD (n = 4) or from untransfected control mice (n = 2) and stained with myeloid cell markers CD11b, Ly6G, and Ly6C and analyzed by flow cytometry. The data represent the myeloid cells from 1 mouse each.
(TIF)

S2 Fig Characterization of the germline transgenic mice. **A.** Murine plasma was collected by tail bleeding and diluted in SDS PAGE loading buffer (1:40 for transiently transgenic HGD mice injected with the “human plasmid” with *APOL1*:*HPR* and 1:10 for homozygous germline transgenic mice expressing baboon *APOL1* and *HPR*). The proteins were separated on a non-reducing SDS PAGE gel and probed by western blot for APOL1 and HPR. A known concentration of recombinant proteins (human APOL1 and haptoglobin for HGD and baboon APOL1 and human haptoglobin for germline transgenic mice) were used as standards to determine the concentration of the respective proteins. Quantitation of the protein band was performed using Image J software. **B.** Germline transgenic mice (heterozygous) producing the TLF proteins APOL1 and HPR and control wild-type mice were infected with 5000 *T*. *b*. *brucei* intraperitoneally and monitored for parasitemia and death. Kaplan-Meier curve showing the survival of the mice (****p < 0.0001; Log-rank test). **C.** Baboon plasma was serially diluted (1:40 to 1:320) and protein were separated on a non-reducing SDS PAGE gel. Murine plasma collected by tail bleeding from targeted germline transgenic mice was serially diluted (Homozygous mice- 1:40–1:320 and Heterozygous mice- 1:20–1:320). Separated proteins were then probed by western blot for baboon APOL1 and HPR. * Proteolyzed APOL1.
(TIF)

S3 Fig Neutrophil gating strategy and frequency in mice post neutrophil depletion. Neutrophils were depleted from mice using 1 mg anti-mouse Ly6G clone 1A8 antibody (1A8) or an isotype IgG2A antibody (Isotype) **A.** Mouse blood (50μl) was collected by tail bleed 24 hours after antibody treatment (time of infection); Mouse ears were collected 10 hours after infection with 1x10^6^ metacyclic promastigotes and processed as described in the Materials and Methods section. The white blood cells were then stained with anti-mouse CD11b PE, anti-mouse GR-1 FITC, and anti Ly6C APC and measured by Flow cytometry using a BD FACSCalibur. Total cells were then sub-gated for CD11b^+^ lineage cells. CD11b^+^ lineage cells were then divided into sub-populations. Neutrophils were identified as the CD11b^+^Ly6G^+^GR1^+^ subpopulation. **B.** Quantification of sub-gated neutrophils (CD11b^+^Ly6G^+^GR1^+^) in blood and ear samples.
(TIF)

S4 Fig *Ex vivo* neutrophil infection. Neutrophils were isolated from C57/B6 mouse bone marrow and infected with CFSE stained metacyclic promastigotes at the ratio of 3 parasites to one neutrophil. **A.** Gating strategy used to count the parasites. **B.** Frequency of parasites in neutrophils at 4 hours post infection in the presence of i. hfHDL and bfHDL, ii. tHDL and bHDL and iii. tHDL (LPS) and bHDL
(TIF)

S5 Fig Binding of *L. major* metacyclic promastigotes to tHDL or bHDL. *L*. *major* metacyclic promastigotes (1x10^6^/ml) were treated with 10 μg/ml of DyLight-488 labelled tHDL and bHDL (blue) or not (red) for 30 min on ice. Fluorescence intensity was quantified by flow cytometry.
(TIF)
