# Supplementary figures and images for: Interplay of Trypanosome Lytic Factor and innate immune cells in the resolution of cutaneous Leishmania infection
Source: PLoS Pathog. 2021 Sep 24;17(9):e1008768. doi: 10.1371/journal.ppat.1008768 (PMC8494325; doi:10.1371/journal.ppat.1008768)

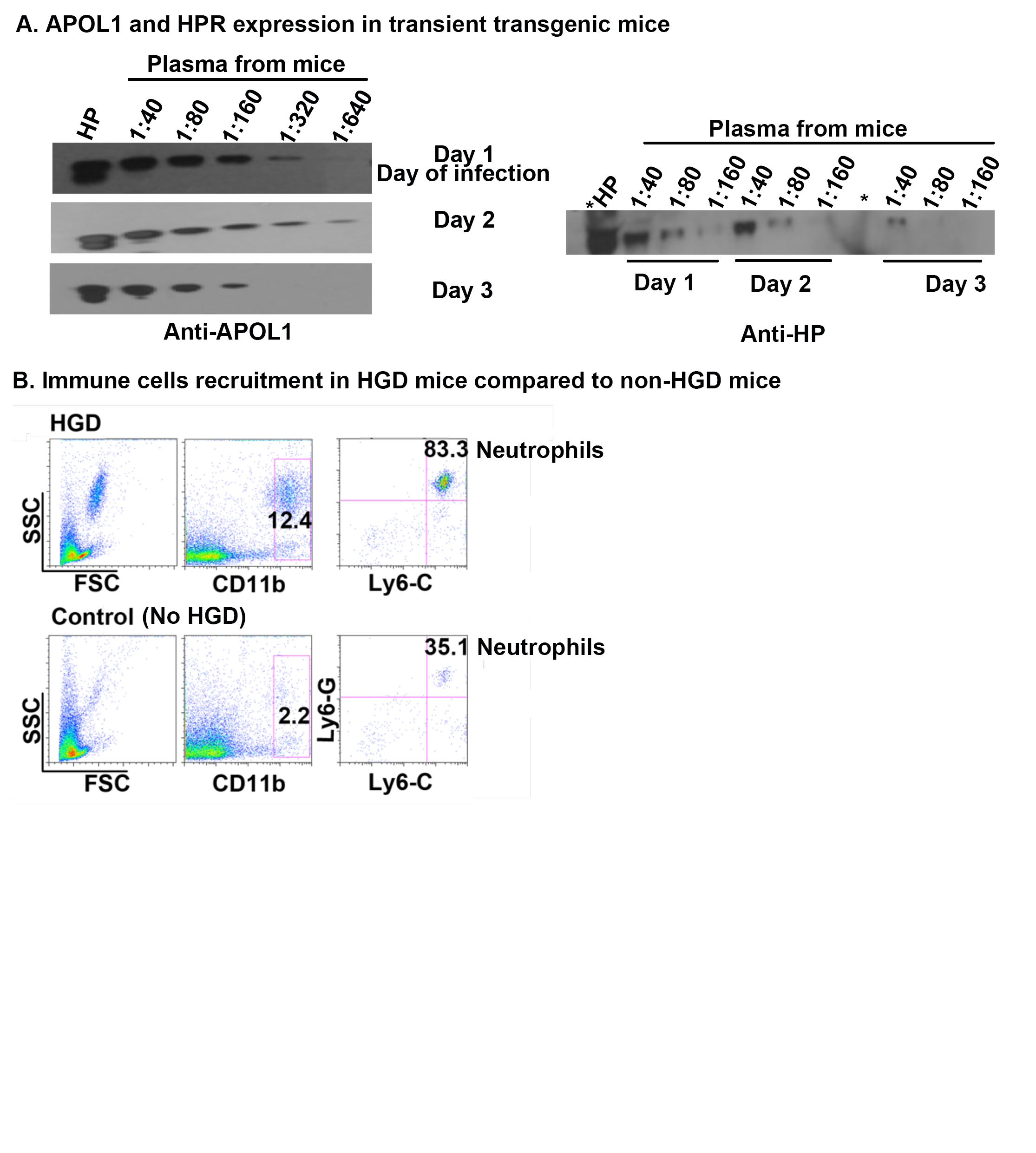

Supplement: S1 Fig — Mice were subjected to HGD and blood was collected A. Plasma from mice collected on 1, 2 and 3 days post HGD were serially diluted and separated by SDS PAGE followed by immunoblotting using anti-APOL1 (for APOL1) and anti-HP (for HPR) antibodies, *HP- Human Plasma. B. Blood was collected 24 hours post-HGD (n = 4) or from untransfected control mice (n = 2) and stained with myeloid cell markers CD11b, Ly6G, and Ly6C and analyzed by flow cytometry. The data represent the myeloid cells from 1 mouse each. (TIF) [file ppat.1008768.s001.tif]

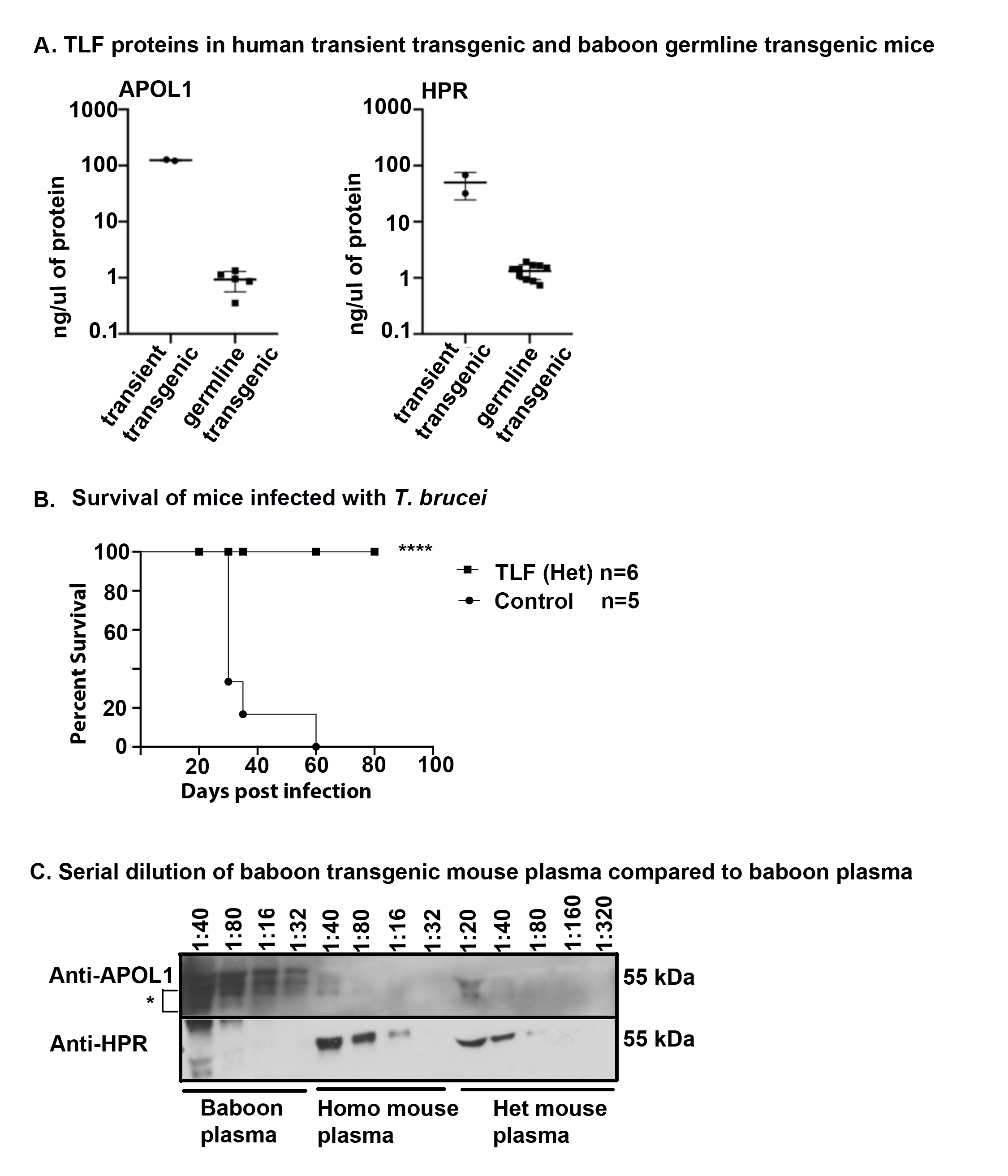

Supplement: S2 Fig — A. Murine plasma was collected by tail bleeding and diluted in SDS PAGE loading buffer (1:40 for transiently transgenic HGD mice injected with the “human plasmid” with APOL1:HPR and 1:10 for homozygous germline transgenic mice expressing baboon APOL1 and HPR). The proteins were separated on a non-reducing SDS PAGE gel and probed by western blot for APOL1 and HPR. A known concentration of recombinant proteins (human APOL1 and haptoglobin for HGD and baboon APOL1 and human haptoglobin for germline transgenic mice) were used as standards to determine the concentration of the respective proteins. Quantitation of the protein band was performed using Image J software. B. Germline transgenic mice (heterozygous) producing the TLF proteins APOL1 and HPR and control wild-type mice were infected with 5000 T. b. brucei intraperitoneally and monitored for parasitemia and death. Kaplan-Meier curve showing the survival of the mice (****p < 0.0001; Log-rank test). C. Baboon plasma was serially diluted (1:40 to 1:320) and protein were separated on a non-reducing SDS PAGE gel. Murine plasma collected by tail bleeding from targeted germline transgenic mice was serially diluted (Homozygous mice- 1:40–1:320 and Heterozygous mice- 1:20–1:320). Separated proteins were then probed by western blot for baboon APOL1 and HPR. * Proteolyzed APOL1. (TIF) [file ppat.1008768.s002.tif]

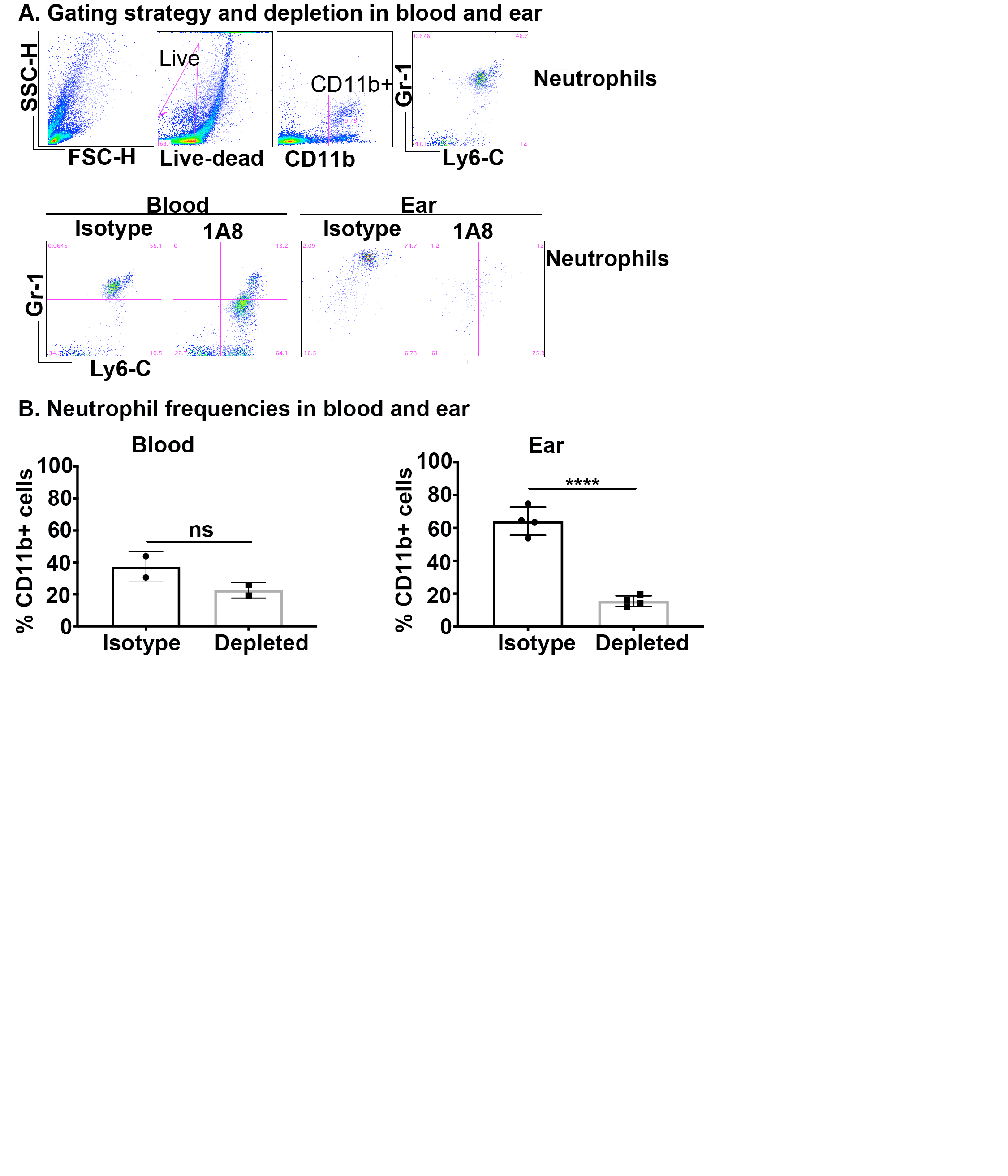

Supplement: S3 Fig — Neutrophils were depleted from mice using 1 mg anti-mouse Ly6G clone 1A8 antibody (1A8) or an isotype IgG2A antibody (Isotype) A. Mouse blood (50μl) was collected by tail bleed 24 hours after antibody treatment (time of infection); Mouse ears were collected 10 hours after infection with 1x10^6^ metacyclic promastigotes and processed as described in the Materials and Methods section. The white blood cells were then stained with anti-mouse CD11b PE, anti-mouse GR-1 FITC, and anti Ly6C APC and measured by Flow cytometry using a BD FACSCalibur. Total cells were then sub-gated for CD11b+ lineage cells. CD11b+ lineage cells were then divided into sub-populations. Neutrophils were identified as the CD11b+Ly6G+GR1+ subpopulation. B. Quantification of sub-gated neutrophils (CD11b+Ly6G+GR1+) in blood and ear samples. (TIF) [file ppat.1008768.s003.tif]

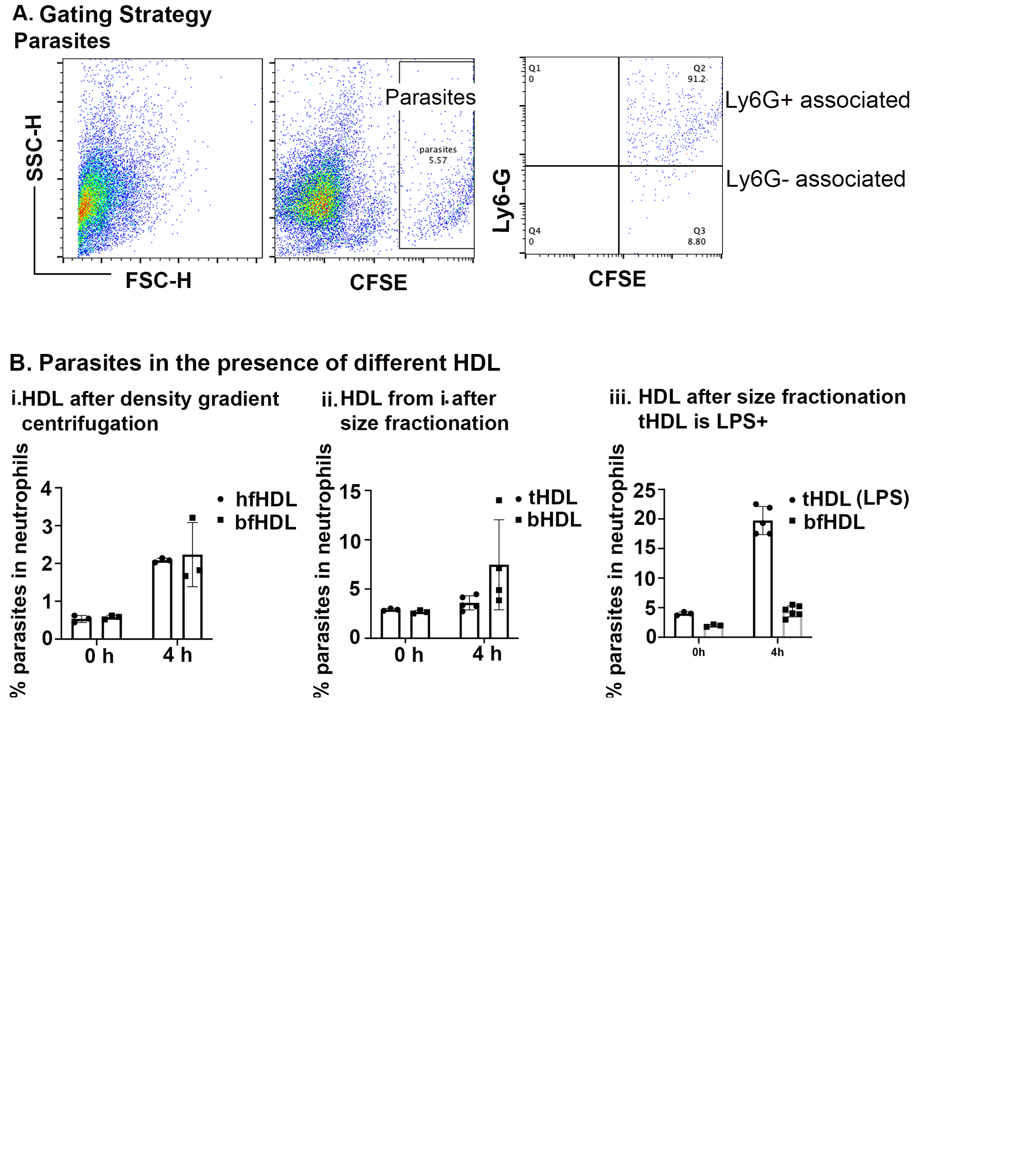

Supplement: S4 Fig — Neutrophils were isolated from C57/B6 mouse bone marrow and infected with CFSE stained metacyclic promastigotes at the ratio of 3 parasites to one neutrophil. A. Gating strategy used to count the parasites. B. Frequency of parasites in neutrophils at 4 hours post infection in the presence of i. hfHDL and bfHDL, ii. tHDL and bHDL and iii. tHDL (LPS) and bHDL (TIF) [file ppat.1008768.s004.tif]

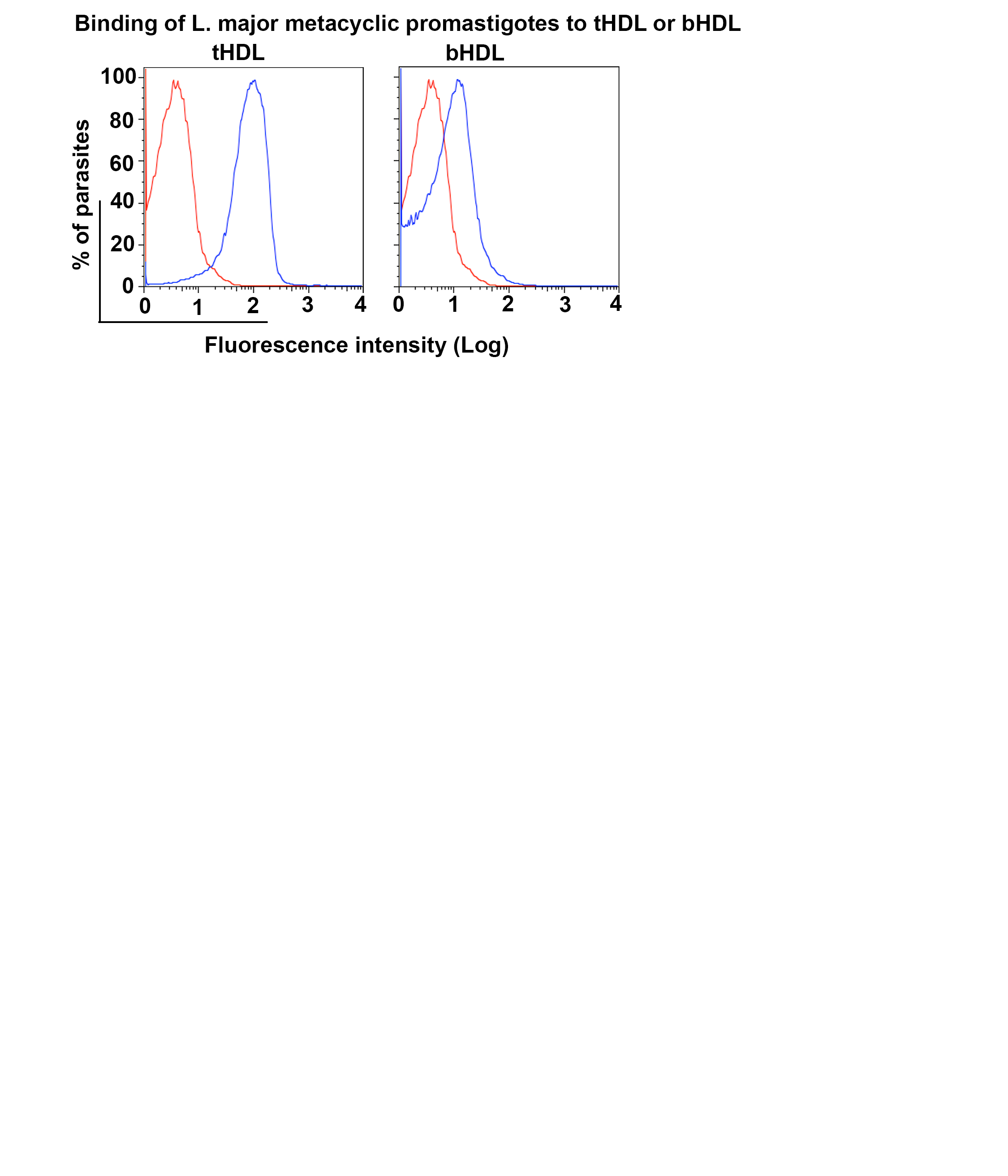

Supplement: S5 Fig — L. major metacyclic promastigotes (1x10^6^/ml) were treated with 10 μg/ml of DyLight-488 labelled tHDL and bHDL (blue) or not (red) for 30 min on ice. Fluorescence intensity was quantified by flow cytometry. (TIF) [file ppat.1008768.s005.tif]
